# Loss-of-Function Mutations in *PTPN11* Cause Metachondromatosis, but Not Ollier Disease or Maffucci Syndrome

**DOI:** 10.1371/journal.pgen.1002050

**Published:** 2011-04-14

**Authors:** Margot E. Bowen, Eric D. Boyden, Ingrid A. Holm, Belinda Campos-Xavier, Luisa Bonafé, Andrea Superti-Furga, Shiro Ikegawa, Valerie Cormier-Daire, Judith V. Bovée, Twinkal C. Pansuriya, Sérgio B. de Sousa, Ravi Savarirayan, Elena Andreucci, Miikka Vikkula, Livia Garavelli, Caroline Pottinger, Toshihiko Ogino, Akinori Sakai, Bianca M. Regazzoni, Wim Wuyts, Luca Sangiorgi, Elena Pedrini, Mei Zhu, Harry P. Kozakewich, James R. Kasser, Jon G. Seidman, Kyle C. Kurek, Matthew L. Warman

**Affiliations:** 1 Department of Orthopaedic Surgery, Children's Hospital Boston and Harvard Medical School, Boston, Massachusetts, United States of America; 2 Howard Hughes Medical Institute, Boston, Massachusetts, United States of America; 3 Department of Genetics, Harvard Medical School, Boston, Massachusetts, United States of America; 4 Division of Genetics, Program in Genomics, and The Manton Center for Orphan Disease Research, Children's Hospital Boston, Boston, Massachusetts, United States of America; 5 Department of Pediatrics, Harvard Medical School, Boston, Massachusetts, United States of America; 6 Division of Molecular Pediatrics, Centre Hospitalier Universitaire Vaudois, Lausanne, Switzerland; 7 Laboratory for Bone and Joint Diseases, Center for Genomic Medicine, RIKEN, Tokyo, Japan; 8 Department of Medical Genetics, Paris Descartes University, INSERM U781, Hôpital Necker Enfants Malades, Paris, France; 9 Department of Pathology, Leiden University Medical Centre, Leiden, The Netherlands; 10 Department of Medical Genetics, Hospital Pediátrico de Coimbra, Coimbra, Portugal; 11 Victorian Clinical Genetics Services, Murdoch Childrens Research Institute, Melbourne, Australia; 12 Department of Pediatrics, University of Melbourne, Melbourne, Australia; 13 Department of Clinical Pathophysiology, University of Florence and Meyer Children's Hospital Genetics Unit, Florence, Italy; 14 de Duve Institute, Université Catholique de Louvain, Brussels, Belgium; 15 Department of Clinical Genetics, Arcispedale S. Maria Nuova, Reggio Emilia, Italy; 16 Merseyside and Chesire Regional Genetics Service, Alder Hey Hospital, Liverpool, United Kingdom; 17 Department of Orthopaedic Surgery, Yamagata University Faculty of Medicine, Yamagata, Japan; 18 Department of Orthopaedic Surgery, University of Occupational and Environmental Health, Kitakyushu, Japan; 19 Department of Pediatrics, S. Anna Hospital, Lugano, Switzerland; 20 Department of Medical Genetics, University of Antwerp, Antwerp, Belgium; 21 Department of Medical Genetics, Rizzoli Orthopaedic Institute, Bologna, Italy; 22 Department of Pathology, Children's Hospital Boston and Harvard Medical School, Boston, Massachusetts, United States of America; University of Oxford, United Kingdom

## Abstract

Metachondromatosis (MC) is a rare, autosomal dominant, incompletely penetrant combined exostosis and enchondromatosis tumor syndrome. MC is clinically distinct from other multiple exostosis or multiple enchondromatosis syndromes and is unlinked to *EXT1* and *EXT2*, the genes responsible for autosomal dominant multiple osteochondromas (MO). To identify a gene for MC, we performed linkage analysis with high-density SNP arrays in a single family, used a targeted array to capture exons and promoter sequences from the linked interval in 16 participants from 11 MC families, and sequenced the captured DNA using high-throughput parallel sequencing technologies. DNA capture and parallel sequencing identified heterozygous putative loss-of-function mutations in *PTPN11* in 4 of the 11 families. Sanger sequence analysis of *PTPN11* coding regions in a total of 17 MC families identified mutations in 10 of them (5 frameshift, 2 nonsense, and 3 splice-site mutations). Copy number analysis of sequencing reads from a second targeted capture that included the entire *PTPN11* gene identified an additional family with a 15 kb deletion spanning exon 7 of *PTPN11*. Microdissected MC lesions from two patients with *PTPN11* mutations demonstrated loss-of-heterozygosity for the wild-type allele. We next sequenced *PTPN11* in DNA samples from 54 patients with the multiple enchondromatosis disorders Ollier disease or Maffucci syndrome, but found no coding sequence *PTPN11* mutations. We conclude that heterozygous loss-of-function mutations in *PTPN11* are a frequent cause of MC, that lesions in patients with MC appear to arise following a “second hit,” that MC may be locus heterogeneous since 1 familial and 5 sporadically occurring cases lacked obvious disease-causing *PTPN11* mutations, and that *PTPN11* mutations are not a common cause of Ollier disease or Maffucci syndrome.

## Author Summary

Children with cartilage tumor syndromes form multiple tumors of cartilage next to joints. These tumors can occur inside the bones, as with Ollier disease and Maffuci syndrome, or on the surface of bones, as in the Multiple Osteochondroma syndrome (MO). In a hybrid syndrome, called metachondromatosis (MC), patients develop tumors both on and within bones. Only the genes causing MO are known. Since MC is inherited, we studied genetic markers in an affected family and found a region of the genome, encompassing 100 genes, always passed on to affected members. Using a recently developed method, we captured and sequenced all 100 genes in multiple families and found mutations in one gene, *PTPN11*, in 11 of 17 families. Patients with MC have one mutant copy of *PTPN11* from their affected parent and one normal copy from their unaffected parent in all cells. We found that the normal copy is additionally lost in cartilage cells that form tumors, giving rise to cells without *PTPN11*. Mutations in *PTPN11* were not found in other cartilage tumor syndromes, including Ollier disease and Maffucci syndrome. We are currently working to understand how loss of *PTPN11* in cartilage cells causes tumors to form.

## Introduction

Cartilage tumor syndromes are characterized by multiple cartilaginous bone tumors that develop in childhood, often causing significant morbidity and predisposing to chondrosarcoma. Tumors can form as exostoses (on the surface of bone), as in the autosomal dominant, multiple osteochondroma (hereditary multiple exostoses) syndromes (MO; MIM 133700 and 133701), or as endosteal tumors (within bone), as in the sporadically occurring multiple enchondromatosis disorders (MIM 166000) Ollier disease and Maffucci syndrome. In MO, mutations in *EXT1* or *EXT2*, which encode heparan sulfate glycosyltransferases, affect chondrocyte orientation in the growth plate [1]. A small percentage of patients with Ollier syndrome have mutations in *PTH1R*, which encodes the receptor for parathyroid hormone and parathyroid hormone-related protein, causing altered chondrocyte differentiation in the growth plate [2]. The cause of Maffucci syndrome is unknown [3]. Patients with MO do not develop endosteal tumors, and patients with Ollier disease or Maffucci syndrome do not develop exostotic tumors [1], [3], [4].

**Figure 1:**
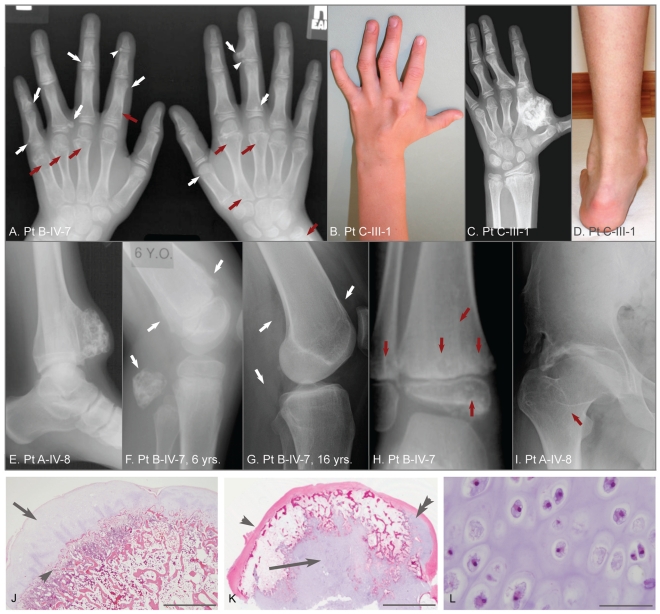
Clinical, radiographic, and histologic features of metachondromatosis. (A) Hand radiographs of participant B-IV-7, taken when 8-years-old. Exostotic lesions (white arrows) are present in the phalanges and metacarpals, and arise from the metaphysis (arrows) or epiphysis (arrowheads). Exostoses tend to point toward the adjacent joint. Endosteal lesions (red arrows) cause metaphyseal expansion. (B, C, D) Hand photograph and radiograph, and foot photograph of participant C-III-1 taken when 9-years-old, depicting mild shortening and deformity of the digits, a large exostotic lesion arising from the second metacarpal bone in the hand, and ankle enlargement superior to the malleoli due to exostoses of the tibia and fibula. (E) Ankle radiograph of participant A-IV-8 taken when 19-years-old depicting a recurrence of a previously excised exostotic lesion of the distal tibia that spans the physis. (F, G) Lateral knee radiographs of participant B-IV-7, taken at 6 years and 16 years, respectively. Note that multiple exostotic lesions of the distal femur and proximal fibula (white arrows) seen when 6-years-old (F) have regressed in the absence of surgical intervention by 16-years of age (G). (H) Ankle radiograph of participant B-IV-7, taken when 5-years-old, demonstrating radiolucency associated with endosteal lesions (red arrows) in the tibia and fibula, and mild metaphyseal flaring. Despite combined metaphyseal and epiphyseal involvement, this individual's linear growth was not affected. (I) Hip radiograph of participant A-IV-8 taken when 22-years-old depicting an endosteal lesion of the femoral neck (arrow) that has caused degeneration of the femoral head and spurring of the acetabulum. (J) Low power photomicrograph of an hematoxylin and eosin (*H&E*) stained section through an exostosis that had been excised from a patient with hereditary multiple exostoses. Note this exostosis is a typical osteochondroma, having a well-developed surface cartilaginous cap (arrow) and endochondral bone immediately below (arrowhead). The scale bar represents 0.15 cm (K) Photomicrograph of an H&E stained exostotic lesion excised from participant A-IV-5 when 5-years-old. This lesion is predominantly covered by a fibrous cap (arrow) and has only a small, eccentric cartilaginous cap (double arrowhead). The majority of cartilage in this, and in 14 other exostoses from patients with MC that have been analyzed, is found within a central core (arrow) and has bone formation occurring at the periphery of this cartilage core. The scale bar represents 0.5 cm. (L) High-power image of the central cartilage core shows chondrocytes with prominent cytoplasm no organization typical of a growth plate. The scale bar represents 100 µm.

Patients with metachondromatosis (MC; MIM 156250) form exostotic and endosteal tumors (Figure 1). Fewer than 50 cases of MC have been published since Maroteaux's initial description in 1971 [5]. Exostotic lesions in MC occur frequently in the digits, involve metaphyses and epiphyses, and tend to grow toward the joint; in contrast, exostotic lesions in MO occur frequently in the long bones, involve only the metaphyses, and tend to grow away from the joint [6]–[11]. MC exostotic lesions can also spontaneously decrease in size and completely regress [6], [7], [9], [12]. Endosteal lesions in MC are common in the metaphyses of long bones and in the pelvis [7]–[11]. Avascular necrosis of the femoral head, due to endosteal tumors, has been a frequent complication in patients with MC [7], [8], [13]–[15]. Hand deformity due to endosteal tumors is uncommon in patients with MC, whereas it is often a significant problem for patients with Ollier disease and Maffucci syndrome [3]. Finally, malignant transformation has only been reported in one patient with MC, whereas it has been more frequently reported in patients with MO, Ollier disease, and Maffucci syndrome [3], [4], [16]. The distinct distribution and clinical behavior of lesions in patients with MC, suggest that MC is pathophysiologically distinct from these other cartilage tumor syndromes. We therefore sought to better characterize MC and to determine its genetic basis.

## Results

### Patient selection

We diagnosed participants as having MC based upon the presence of both multiple exostotic and endosteal cartilaginous lesions as previously described [5]–[10], [15]. We excluded from analysis participants with solitary lesions, contiguous endosteal lesions suggestive of Ollier disease, soft tissue lesions suggestive of Maffucci syndrome, or radiographs suggestive of MO. We included participants who had clinical and radiographic features of MC, even if they lacked a positive family history. For each patient, the clinical history and radiographs were reviewed by at least 3 authors. MC patients from 17 unrelated families from 9 countries were identified (Supplementary Table 1). All participants gave their informed consent following the guidelines of each referring institution. In 10 families disease segregation is consistent with autosomal dominant inheritance. In 7 families, the disease is suspected to have arisen *de novo*. Immediate family members of patients with sporadically occurring MC were interviewed and examined, although detailed imaging was not performed. For 8 familial cases, blood or DNA was available from additional family members.

### Clinical and pathologic features of metachondromatosis

Figure 1 depicts features seen in affected participants with MC. No phenotypic differences could be found between sporadic or familial cases of MC. Radiographs identify exostotic and endosteal lesions of the digits (Figure 1A–1C) and long bones (Figure 1E–1H), along with degenerative hip disease secondary to endosteal lesions in the femoral neck (Figure 1I). Spontaneous regression of exostotic lesions is seen in radiographs obtained 10 years apart in the same patient (Figure 1F,1G). Also depicted in Figure 1 are histopathologic features that distinguish exostoses in patients with MO from those in patients with MC, based upon a comparison of 30 exostoses excised from children with MO and 15 exostotic lesions excised from 3 affected individuals with MC. Exostoses in children with MO have cartilage caps with endochondral bone growth immediately beneath the cap (Figure 1J). In contrast, exostoses in children with MC have a predominantly fibrous cap and a core of disorganized cartilage surrounded by trabecular bone (Figure 1K, 1L). In all MC cases, the lesions were bilateral and not obviously confined to a single body segment as in Ollier or Maffucci patients.

### Linkage analysis

**Figure 2:**
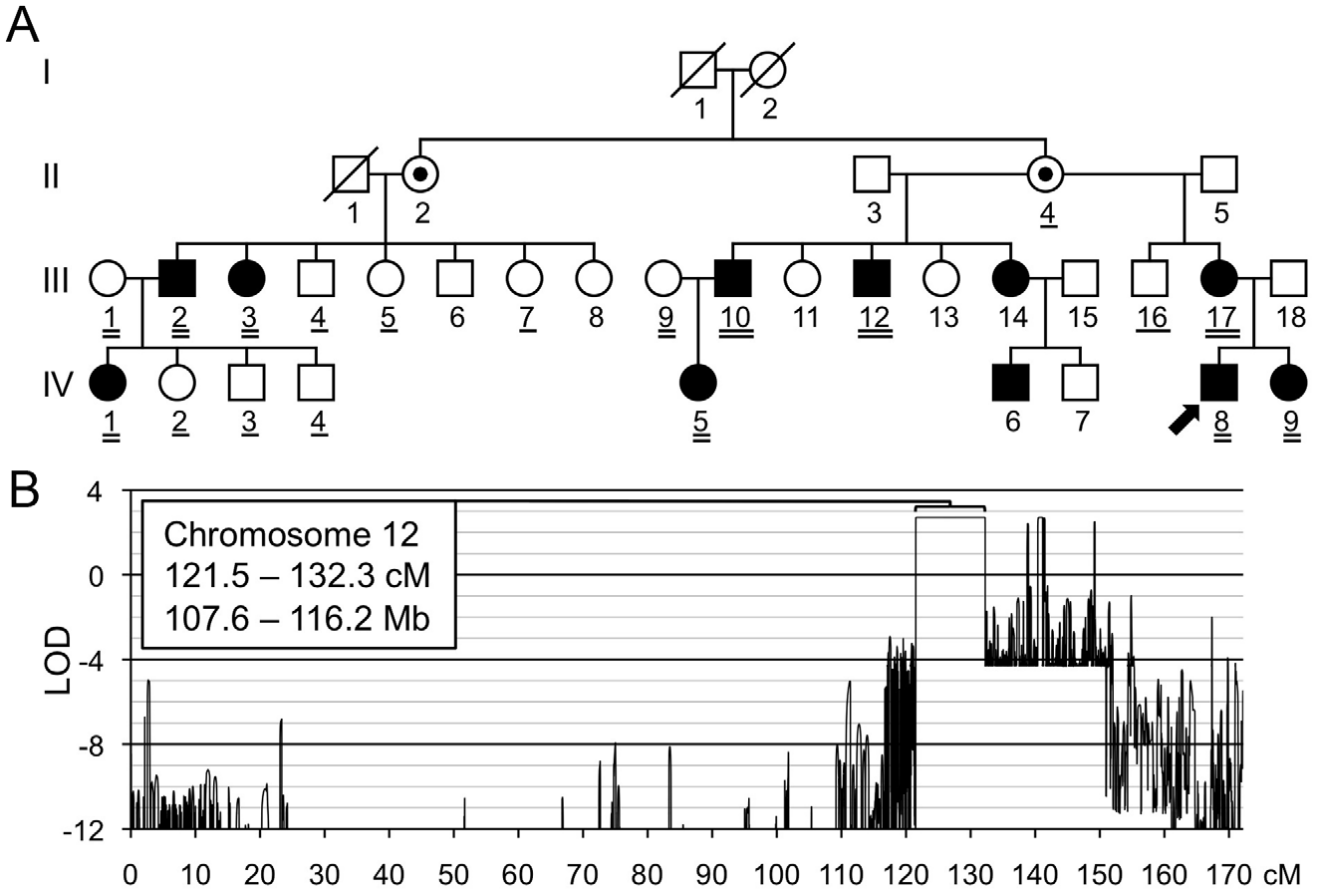
Linkage mapping of metachondromatosis to chromosome 12q. (A) Pedigree of the family (Family A) used to define the metachondromatosis candidate interval. Affected individuals have filled symbols. Individuals who were not examined but who were assumed to be obligate carriers have interior dots. An arrow identifies the proband. Participants whose DNA was used for linkage analysis are double underlined. Those participants with a single or double underlines were tested for the *PTPN11* mutation, after the mutation had been identified in participants A-III-10 and A-IV-5 by targeted array capture and Illumina sequencing. (B) LOD score plot of chromosome 12. Only one interval, larger than 1 cM, in the entire genome attained the maximum LOD score of 2.7. Several autosomal intervals, each smaller than 1 cM, also achieved maximum or positive LOD scores (Figure S1). These likely represent either genotyping errors or short ancestral haplotypes that are coincidental with linkage. The physical coordinates shown are derived from GRCh37/hg19.

We performed linkage analysis in the largest family (Family A, Figure 2A) to identify a genetic locus for MC. Raw genotype data were generated using Affymetrix 6.0 SNP arrays and multipoint parametric linkage analysis of the autosomal genome was performed using MERLIN [17]. Because non-penetrance and non-ascertainment are potential confounding factors in the diagnosis of MC, we analyzed only founders and affected individuals (Figure 2A). Although this limited the maximum attainable LOD score to 2.7, which is lower than the genome-wide significance threshold of 3.3, the dense marker set ensured a reasonable probability that only one large interval that achieved the maximum LOD score would be observed, with the remainder of the genome being excluded.

We identified a single interval on chromosome 12, from 121.5 to 132.3 cM, that attained the maximum LOD score of 2.7 (Figure 2B). No other autosomal interval >1 cM yielded a peak LOD score >−1.9. Several intervals <1 cM attained LOD scores >0 (Figure S1), but we considered these unlikely to be candidate intervals and instead assumed they represented either unfiltered genotyping errors or short ancestral population haplotypes, rather than familial haplotypes inherited from a common ancestor.

### Multiplexed targeted genomic capture and sequencing of linkage interval

We next performed array-based capture, followed by Illumina GAII sequencing, using bar-coded DNA libraries created from 16 individuals in 11 families. We sequenced 1 affected individual in 8 families, 2 affected individuals in 1 family, and 2 affected and 1 unaffected individual in 2 families (Table S1). We prepared bar-coded genomic DNA libraries, having an average insert size of 150 bp, using sheared DNA from each of the 16 individuals (Figure S2). We performed array-based targeted capture by pooling each DNA library and hybridizing the pooled sample to an Agilent Technologies 1M SureSelect DNA capture array containing 973,952 probes targeting 844,339 bp within the 8.6 Mb candidate interval, including 88.4% and 98.6% of UCSC exons and CCDS coding sequence, respectively. After hybridization and elution, the captured DNA was PCR amplified, purified to remove primer dimers, and sequenced on two lanes of an Illumina GA II.

We obtained 50 million, 80 bp, single-end reads. Novobarcode software was used to sort the reads according to their 3 bp barcode, and Novoalign was used to align the reads to the reference genome (hg19). We obtained between 1 and 6 million reads for each individual. Among individuals, an average of 61% (±2%) of the aligned reads mapped to regions targeted by the capture array (Figure S3A). Of the bases targeted by the capture array, 75% (±7%) had a read depth of at least 5×, which diminished to 55% (±14%) after the removal of PCR duplicates (Figure S3B). Twenty percent of targeted bases were not captured. In the 3 families for which pairs of affected family members were sequenced, total filtered variants in the candidate interval (388–1499 per individual) were analyzed to find variants shared by both affected individuals from the same family (Table 1). In all 3 families, frameshift mutations in exon 4 of *PTPN11* were the only novel coding variants present in both affected family members and, for Families A and B, absent in the unaffected individual. Family A had a 5 bp deletion, Family B had a more complex deletion/insertion, and Family C had a 2 bp deletion (Table S1). In the remaining 8 families for which only 1 affected individual per family was sequenced, there were 18 novel coding variants present in ≥3 reads, one of which was a nonsense mutation in exon 13 of *PTPN11* (p.Q506X) (Figure S4). We used Sanger sequence analysis of PCR amplimers to demonstrate that affected family members from these 4 families had *PTPN11* mutations, and that unaffected family members lacked *PTPN11* mutations.

**Table 1:** Novel coding variants identified in three metachondromatosis families.

Family	A		B		C	
Individual	III-10	IV-5	IV-7	III-5	III-1	II-5
Total variants*	529	388	536	1499	480	709
Coding	45	40	50	200	37	51
Not listed in dbSNP	6	12	10	167	5	7
Shared	3		1		1	
Not present in unaffected family member	1		1		n/a	
Genes affected and predicted protein changes	***PTPN11*** p.V137fs		***PTPN11*** p.T153fs		***PTPN11*** p.S118fs	

*filtered to remove low confidence variants.

Sanger sequence analysis of the 15 coding exons of *PTPN11* in the 7 families for whom we had not found mutations by array capture and Illumina-sequencing detected a 1 bp deletion in exon 11 in 1 of the 7 families (Family D). This deletion was within a 98 bp segment that had been targeted but not captured in any of the DNA samples. Another family (F) had a splice-acceptor site mutation (AG>CG) in intron 5 in 2 affected siblings, but not in either parent. The siblings' mother was clinically affected with MC, although less severely than her children. The mother was the first in the family to have MC and was the only member of the family who was included in the Illumina sequencing. The site of the splice-site mutation identified in her children was covered 25× in her DNA sequence and was always wild-type, as were her Sanger sequence results. These data suggest the mother is mosaic for a *PTPN11* mutation and that the family's mutation would have been found by Illumina sequencing had we initially sequenced her children's DNA.

**Figure 3:**
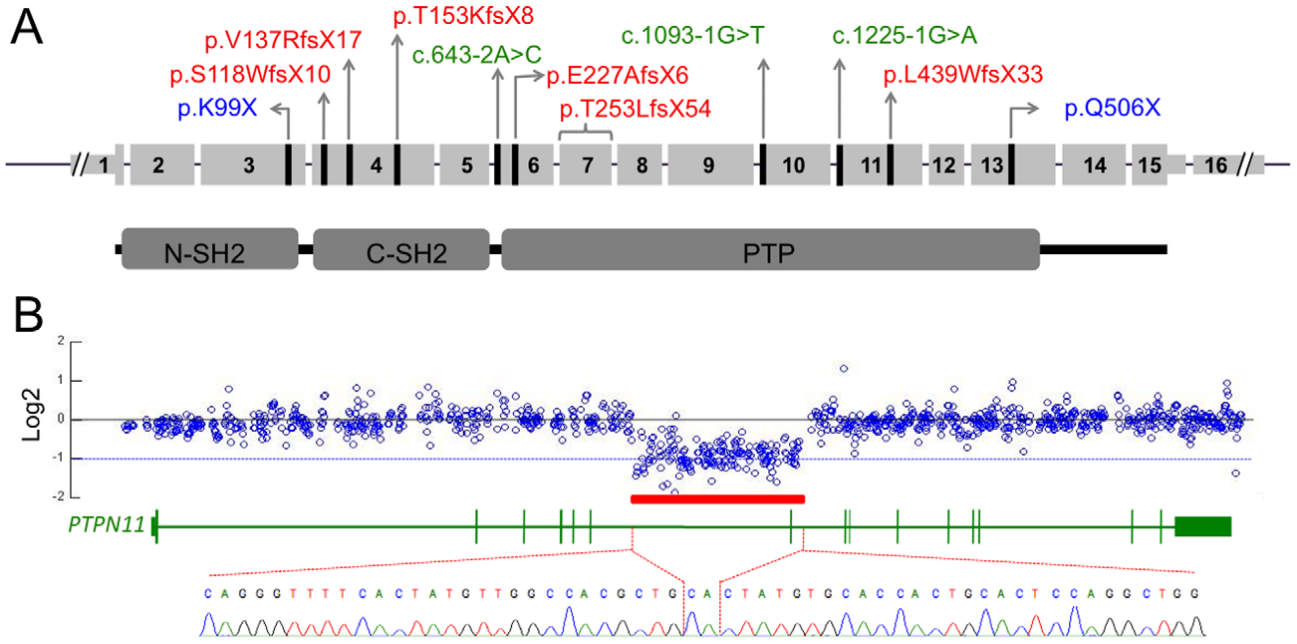
*PTPN11* mutations identified in MC participants. (A) Schematic of the exonic structure of *PTPN11* (above) and the corresponding protein structure of SHP2 (below). The locations of mutations that were identified in MC are indicated with black lines. Predicted protein changes are indicated for the nonsense (blue) and frameshift (red) mutations, while the cDNA designation is indicated for the splice-site mutations (green). (B) Log2 values of the number of Illumina reads obtained per 50 bp window in participant S, divided by the average number of reads obtained in other participants whose DNA was captured simultaneously using the second capture array. Shown are all 50 bp windows spanning regions of *PTPN11* targeted by the array, with the corresponding exonic structure of *PTPN11* shown below. The red bar indicates a region spanning exon 7, in which the average log2 value is approximately −1, suggesting a heterozygous deletion. PCR amplification and sequencing of the breakpoint, using primers on either end of the deletion, indicate that 14,629 bp of sequence have been deleted and replaced with a single CA dinucleotide.

We subsequently collected DNA from an additional 6 MC families. Sanger sequence analysis revealed a nonsense mutation involving exon 3 (p.K99X) in one family (I), and a splice site mutation in intron 9 (c.1093-1G>T) in another family (G). In total, we found *PTPN11* mutations in 10 of 17 families. Five mutations were frameshift, 2 were nonsense, and 3 disrupted a splice-acceptor site (Figure 3A, Table S1). Each family had a different mutation and mutations were scattered across the gene (Figure 3A). In two families without mutations we had performed aCGH and did not find evidence of *PTPN11* intragenic deletions or duplications (Table S1). We did not have other family members' DNA samples from the one familial MC patient who lacked a *PTPN11* mutation to be able to test for locus heterogeneity by linkage analysis.

### Multiplexed targeted genomic capture and sequencing of *PTPN11* and associated genes

To test whether the patients without *PTPN11* coding mutations had noncoding mutations in *PTPN11* or had mutations in other genes, we designed a second Agilent 1M capture array. Firstly, we included probes to target the entire *PTPN11* gene, excluding Alu repeats. Secondly, we included probes targeting the exons of 74 genes that function in the same pathways as *PTPN11*, including the Ras/MAPK and PI3K/Akt pathways (Table S2). Thirdly, we included probes targeting the exons of the MO genes *EXT1* and *EXT2*, to determine whether any of our patients lacking mutations and classic radiographic features of MC had been misdiagnosed. Barcoded genomic libraries for an individual from each of the 7 MC families without *PTPN11* coding mutations and from 2 families originally referred with MC, but whose radiographic features were more consistent with MO, were pooled and hybridized to the capture array. The captured DNA was then sequenced using two lanes of Illumina GAII 42 bp single end sequencing. For each barcoded sample, 4.3 (±1.2) million reads were obtained, of which 37% (±7%) mapped to regions targeted by the array (Figure S3A). Of the bases targeted by the array, 85% (±6%) were covered by a read depth of at least 10×, which dropped to 83% (±8%) after the removal of PCR duplicate reads (Figure S3B).

Identified variants with a quality score >20 were filtered to remove SNPs listed in the SNP database (version 132) and the 1000 genomes project (Nov. 2010 release). No *PTPN11* coding mutations were found. We then analyzed the noncoding regions and identified one 3′ UTR mutation, and 7 intronic mutations, of which 6 were in LINE elements or other repetitive regions (Table S3). All intronic mutations were at least 300 bp from the nearest exon and, using an online splice prediction tool (http://genes.mit.edu/GENSCAN.html), were not predicted to alter splicing. We then analyzed variants identified in the exons of the 76 other genes included in the capture array (Table S2). No nonsense, frameshift or splice site mutations were identified. Of the missense mutations that were present in more than 4 independent sequencing reads, 4 were nonsynonomous (*ERBB2* p.S1050L, *MTOR* p.P1408S, *MVP* p.R49S, *SOS2* p.D952N) and 4 were synonomous (*PIK3C2B* p.D478D, *RAF1* p.L351L, *MAP2K2* p.D140D, *MVP* p.T199T). Further experiments will be needed to determine if any of the novel noncoding *PTPN11* mutations or novel variants in the other genes are disease causing.

We next analyzed the sequencing read depth across the *PTPN11* locus to detect deletions or duplications. In one individual (Patient S), we identified an ∼15 kb region spanning exon 7 that contained half as many reads as would be expected based upon the read depths of the other patients included in the capture array (Figure 3B). As expected, PCR primers that flank this 15 kb region failed to produce amplimers when wild-type genomic DNA was used as template. However, PCR amplification using genomic DNA from Patient S yielded an ∼700 bp PCR product and Sanger sequence analysis of this product indicated that 14,629 bp of genomic sequence (chr12:112,897,487–112,912,115) had been replaced with a single CA dinucleotide (Figure 3B). In addition, PCR amplification and sequencing of *PTPN11* in peripheral blood cDNA from this patient, using a forward primer in exon 6 and a reverse primer in exon 8, detected a mutant cDNA that lacked exon 7 (data not shown). The loss of exon 7 results in a frameshift with introduction of a premature stop codon (T253LfsX54).

The read depth of the remaining 76 genes targeted by the array was also analyzed to detect deletions or duplications. Two patients initially included in the study, but on radiographic review were felt more likely to have MO than MC, were found to have deletions involving *EXT1* (Figure S5). In one patient (Q), the first exon of *EXT1* contained half as many reads over its 1.8 kb as expected (Figure S5A). In a second patient (N), all exons of *EXT1* had half as many reads as expected (Figure S5A). In additional to skeletal lesions, this patient has developmental delay, microcephaly and mild dysmorphism, suggesting a possible contiguous gene deletion syndrome. *EXT1* deletions were confirmed in both patients by multiplex ligation-dependent probe amplification (MLPA) (Figure S5B, S5C).

### *PTPN11* loss-of-function mutations in metachondromatosis

Our finding of nonsense, frameshift, and splice-site mutations in multiple exons, as well as a large deletion, suggests that MC-causing *PTPN11* alleles are loss-of-function. We tested this hypothesis by performing Western blots on whole protein extracts from white blood cells and from an excised exostotic lesion in a patient (B-IV-7) with a *PTPN11* frameshift mutation in exon 4. An anti-SHP2 antibody that recognizes an epitope amino-terminal of the polypeptide encoded by the frameshifted exon detected only full-length, wild-type SHP2 protein (Figure S6).

### Loss of *PTPN11* wild-type alleles in the cartilage cores of MC exostoses

**Figure 4:**
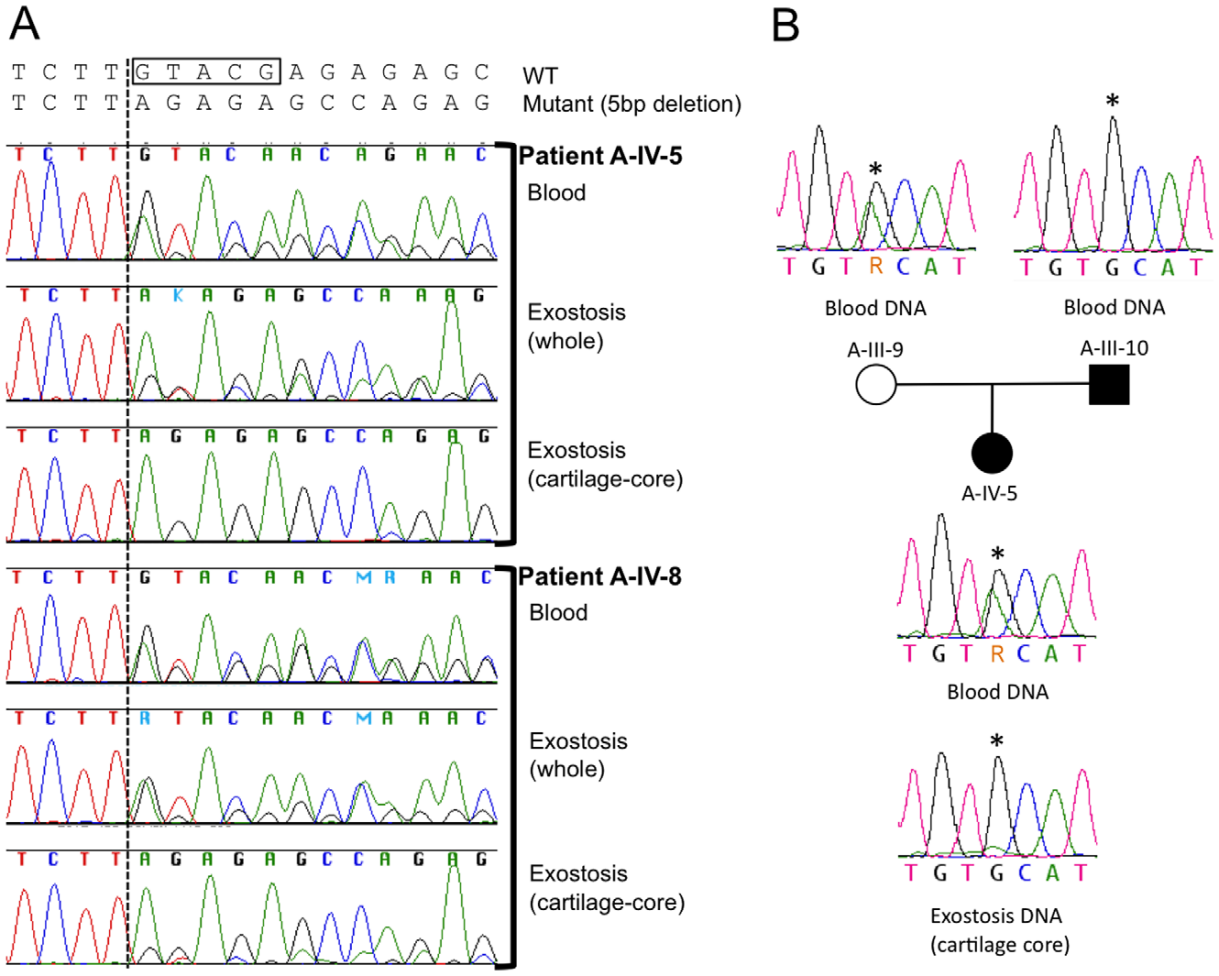
Loss of the wild-type *PTPN11* allele in the cartilage of two exostoses. (A) Electropherograms of PCR amplified template DNA that had been extracted from whole blood, a section of an exostosis, or the cartilage core of the same exostosis. Exostoses were available from patients A-IV-5 and A-IV-8. The site of the 5 bp deletion in exon 4 of *PTPN11* in both patients is indicated with a box. Note that that the heights of the peaks corresponding to the mutant sequence are markedly reduced in amplimers from the cartilage-core compared to amplimers from blood or from a section that contains cartilage, bone and fibrous tissue. This is consistent with loss-of-heterozygosity in the cartilage component of the exostoses. (B) Electropherograms of PCR amplified template DNA extracted from blood from participants A-III-9, A-III-10 and A-IV-5, as well as DNA extracted from the cartilage core of the exostosis from participant A-IV-5 shown in (A). Blood DNA electropherograms indicate that participants A-III-9 and A-IV-10 are heterozygous at a position (asterisk) in intron 11 of *PTPN11*. This is the site of a known common polymorphism (rs41279092). Exostosis cartilage DNA electropherograms have a reduced adenine peak height at this position. This suggests that the wild-type *PTPN11* allele inherited from the unaffected parent (A-III-9), which carries an adenine at this position, has been lost in cells that contribute to formation of the exostosis' cartilage core.

We next determined whether MC exostoses arise from a “second hit,” similar to what has been observed in autosomal dominant MO [18]. We looked for a second hit in cells of the cartilage core of an MC lesion (e.g., Figure 1K) by performing microdissection, PCR amplifying the mutation containing exon, and Sanger sequencing the amplimers. In tumors from two different patients (A-IV-5, A-IV-8), with a 5 bp frameshift mutation in exon 4, we observed a clear excess of mutant sequence versus wild-type sequence in the tumors' cartilage cores, as compared to the patients' peripheral blood and bone/marrow from the lesion (Figure 4A). We quantified the amount of mutant versus wild-type sequence, by extracting DNA from the cartilage core of patient A-IV-8, PCR amplifying exon 4, and subcloning amplimers to determine the percent that contained the mutant allele. Forty-four of 52 individual subclones contained the mutant allele, which is significantly higher (p<0.001) than expected for a heterozygous mutation. In contrast, 58% of subclones (34/59) from adjacent unaffected bone/bone marrow contained the mutant allele, which is not significantly different from the expected value of 50% (p = 0.24). These data are consistent with an MC exostosis arising from a second hit (loss of the wild-type allele) within a cell that ultimately contributes to the lesion's cartilage core.

Because the mutant allele is 5 bp shorter than wild-type PTPN11 in these two patients, we tested for loss of heterozygosity at a second polymorphic site in *PTPN11* to control for potential PCR bias in amplifying the exon with the deletion. In their peripheral blood DNA, patient A-IV-5 and her unaffected mother are heterozygous for a benign polymorphism in intron 11 of *PTPN11* (rs41279092). The abundance of this SNP, which is in the wild-type PTPN11 allele, was markedly reduced in the lesion's cartilage core, again consistent with LOH occurring in the cell that drives formation of the cartilage core (Figure 4B).

### PTPN11 mutations are not found in other cartilaginous tumor syndromes

We finally asked whether mutations in *PTPN11* are associated with other cartilaginous tumor syndromes. We sequenced the coding exons of *PTPN11* in 38 lesions excised from patients with Ollier disease, 2 peripheral blood samples from patients with Ollier disease, 15 lesions excised from patients with Maffucci syndrome, 4 solitary enchondromas, 9 chondrosarcomas (1 polyostotic), and 3 osteochondromas without *EXT1 or EXT2* mutations. We did not find *PTPN11* coding sequence mutations in any patient sample. In 24 percent of the samples we observed heterozygosity for noncoding SNPs that are known common variants, suggesting that large *PTPN11* gene deletions and other causes of LOH are not frequently associated with these other cartilaginous tumors.

## Discussion

We identified 17 unrelated families with MC. Clinical features were similar to previously published cases [5]–[10], [15]. The exostoses of MC had been assumed to be identical to the osteochondromas of MO; however, we demonstrate that they are histologically unique lesions with a large cartilaginous core (Figure 1J–1L). We combined linkage analysis in a single MC family with DNA capture and parallel sequencing of bar-coded DNAs from several MC families to identify mutations in *PTPN11* as a cause of MC. In MC patients without *PTPN11* coding sequence and splice site mutations, we generated and pooled barcoded DNAs, and performed a second targeted capture that included the entire *PTPN11* gene and exons from 76 other genes. This led us to detect an ∼15 kb deletion in a patient by analyzing the depth of sequencing reads (Figure 3B). In total, we found likely disease-causing *PTPN11* mutations in 11 of 17 families.

Concurrent with our studies of MC, Sobreira et al. (2010) reported *PTPN11* mutations in 2 MC families [11]. They performed whole-genome sequencing (WGS) in a single affected individual who was a member of family in which MC was segregating. This approach also required these investigators to include linkage data to reduce the number of novel potentially disease-causing heterozygous changes that are identified by WGS [19], [20]. The investigators next identified an independently arising *PTPN11* mutation in an unrelated patient to strengthen the evidence for causality. However, having only studied genomic DNA and finding frameshift mutations in the same exon (exon 4) in their two unrelated patients, Sobreira et al. (2010) could not definitively determine the mechanism by which *PTPN11* mutations cause MC.

Missense mutations in *PTPN11* have previously been identified in patients with Noonan, Noonan-like, and LEOPARD syndromes, as well as in juvenile myelomonocytic leukemia [21]. In these disorders, the mutations are gain-of-function and/or dominant negative for SHP2, which is the *PTPN11* protein product [22], [23]. SHP2 is a protein tyrosine phosphatase and an important intracellular signaling molecule linking several growth factor receptors to the Ras/MAPK and other signaling pathways (Reviewed in [24]). Therefore, frameshift mutations in exon 4 might also create an abnormal protein product by altering *PTPN11* mRNA splicing. Alternatively, the frameshift mutations might result in loss-of-function because of nonsense mediated mRNA decay or rapid degradation of a truncated SHP2 polypeptide. Our finding of nonsense, frameshift, and splice-site mutations in multiple exons, as well as a whole-exon deletion, suggests that MC-causing *PTPN11* alleles are loss-of-function. We tested this hypothesis by performing Western blots on whole protein extracts from white blood cells and from an excised lesion containing affected and unaffected tissue, and detected only full-length, wild-type SHP2 protein (Figure S6), confirming that the mutant alleles are loss-of-function.

Exostoses in MO originate from the “second hit” mutations [18]. Mice with conditional alleles at the *EXT1* locus demonstrate that only a few cells having two mutant alleles are sufficient to cause other cells to become misdirected and form an exostosis [25]. By performing microdissection, we found evidence for loss of the wild-type *PTPN11* alleles in the majority of cells within the cartilage cores of exostoses from two MC patients (Figure 4), consistent with a “second hit.” Recently, Bauler et al. used a ubiquitously expressed *Ert2-Cre* driver in 6–8 week-old *Ptpn11* floxed mice to generate mice that were *Ptpn11*-null in multiple tissues. Among the consequences of completely deleting SHP2 was the appearance of ectopic cartilage islands in the animals' metaphyseal trabecular bone and growth plates [26]. These findings are consistent with the distribution of endosteal tumors and exostoses seen in patients with MC. The findings in mice with homozygous deletion of *Ptpn11* contrast with the absence of skeletal lesions in mice that have heterozygous loss-of-function mutations [27]. We suspect that mice with heterozygous mutations have a much lower incidence of noticeable “second hits” compared to humans because they have fewer skeletal cells and shorter lifespans. Homozygous inactivation of *Ptpn11* solely in mouse chondrocytes may be required to enable a detailed understanding of how SHP2 deficiency leads to tumorigenesis.

We did not detect *PTPN11* mutations in 6 of 17 patients with MC phenotypes, including 1 patient with a family history of MC and 5 patients who are the first affected members in their families. DNA is not available from other affected family members of the familial case to determine whether MC exhibits locus heterogeneity. Two patients with *de novo* disease did have DNA variants found in the 3′ UTR and/or in introns. None of these variants are in likely regulatory regions or in regions important for mRNA splicing; however, we cannot conclude they are benign. Furthermore, we cannot exclude the possibility that patients with *de novo* disease are somatic cell mosaics for *PTPN11* mutations that are not present in white blood cell DNA, similar to the mildly affected mother in Family F who had two affected children. Despite these caveats, MC could be locus heterogeneous, similar to Noonan syndrome, which can be caused by mutations in *PTPN11* or in other components of the Ras/MAPK pathway [28]; however, our targeted capture and sequencing of 74 genes that included most of the Ras/MAPK and PI3K/Akt signaling pathways did not find an obvious mutation in another gene in any of the 6 *PTPN11* mutation-negative MC patients.

We found no evidence of *PTPN11* coding mutations in other cartilage tumor syndromes, including Ollier disease and Mafucci syndrome. Although sequencing was performed on lesional tissue rather than whole blood, it is possible that we may have missed causative mutations that are present in only a subset of cells within the lesion. We may also have missed mutations in the 5′ and 3′ untranslated regions of *PTPN11* contained within exons 1, 15, and 16, that we did not sequence in these patients. Based on our finding heterozygosity for noncoding SNPs in many of these samples, it is unlikely that large *PTPN11* gene deletions or other causes of LOH are common in these syndromes. Despite the aforementioned limitations of our mutation detection method, our data are consistent with the separation of MC from the other cartilage tumor syndromes based on clinical and pathologic features.

In conclusion, we combined linkage analysis in a single family with DNA capture and parallel sequencing of bar-coded DNAs from several families to identify mutations in *PTPN11* as a cause of MC. The advantages of this approach are its ability to identify a region of interest, then simultaneously sequence affected individuals from multiple unrelated families, and then focus on genes for which novel SNPs or other mutations are seen in more than one family, all at reasonable cost (∼$10,000 in consumables). In patients with MC and *PTPN11* mutations, we conclude that the mutations are loss-of-function since the mutant protein is not expressed, and that the loss of the remaining wild-type allele via a “second hit” is responsible for the formation of the exostoses. Since we did not detect *PTPN11* mutations in all MC families, MC may be locus heterogeneous, although we have not found evidence after sequencing more than 70 genes that function in related pathways. Finally, precisely how mutations in *PTPN11* give rise to the exostoses and endosteal tumors in patients with MC is not yet known. However, this question can now be addressed since mice with alleles of *Ptpn11* that can be conditionally inactivated in temporal and site-specific manner are available [26], [29].

## Materials and Methods

### Ethics

Informed consent was obtained through a Children's Hospital Boston IRB approved protocol. Specimens and/or DNA received from external institutions were collected under IRB approved protocols at host institutions and received coded without identifying information.

### Linkage analysis

Raw genotype data were generated for multiple members of family A using Affymetrix 6.0 SNP arrays, and genotypes were called using Affymetrix Genotyping Console with the Birdseed v2 algorithm and a confidence threshold of 0.02. SNPs with <100% sample call rate or pedigree minor allele frequency of 0 were removed, then multipoint parametric linkage analysis of the remaining 421,922 autosomal and 17,169 X-linked SNPs was performed using MERLIN and its derivative MINX, respectively, with Affymetrix Caucasian allele frequencies and deCODE Genetics genetic map positions. The disease allele frequency was estimated at 1E-7, and phenocopies and non-penetrance were not permitted (affectation probability 0/1/1). Because non-penetrance and non-ascertainment are potential confounding factors in MC, we analyzed only founders and affected individuals.

### Barcoded genomic libraries

To generate genomic libraries for each individual, 2 µg of genomic DNA were first sheared to ∼100 bp–200 bp using Adaptive Focused Acoustics following the manufacturer's protocol (Covaris, Inc). Blunt-ended fragments were generated using an End-it DNA End-Repair kit (Epicenter), purified using Agencourt AMPure XP magnetic beads (Beckman Coulter), and eluted in 10 mM Tris Acetate, pH 8.0. The fragments were A-tailed using the Klenow fragment (NEB), purified, eluted in 1× Quick Ligase Buffer (Quick Ligation kit, NEB), and incubated with Quick T4 DNA ligase and 100 µM barcoded-adapters (Table S4) to create a library of adapter-ligated fragments. A different barcoded adapter was used for each genomic DNA library. Each library was again purified using Agencourt AMPure XP magnetic beads and eluted in 40 µl of 10 mM Tris Acetate, pH 8.0. Libraries used for hybridization to the first capture array were amplified according to two strategies: 3 µl amplified for 18 cycles in four 50 µl PCR reactions (Phusion High-Fidelity DNA polymerase, Finnzymes), or 2 µl amplified for 11 cycles in one 50 µl PCR reaction that was then purified and amplified for 17 cycles in ten 50 µl PCR reactions (FastStart Taq DNA polymerase, Roche). For the libraries used for hybridization to the second capture array, 13 µl was amplified for 15 cycles in ten 50 µl PCR reactions (Phusion High-Fidelity DNA polymerase, Finnzymes). Primers are provided in Table S5. Sizes of amplified libraries were confirmed to be between 200–300 bp necessary for Illumina GA II sequencing prior to hybridization (Figure S2).

### Capture array design

To enrich regions of interest in the linked interval for sequencing, we used an Agilent Technologies 1M SureSelect DNA capture array. Target regions were defined using the UCSC Genome Browser and included: the union of exons from multiple GRCh37/hg19 gene, mRNA, and Alt Events tracks; 30 bp of proximal and distal intronic flanking sequence; and 1000 bp of upstream promoter sequence. Targets were padded with 60 bp of additional proximal and distal flanking sequence to promote uniform capture coverage, for a total size of 1,187,477 bp. Probes were designed against NCBI36/hg18 using the Agilent eArray software (https://earray.chem.agilent.com/earray/) and translated coordinates, with 60-nt length, 3-nt spacing, and repetitive elements masked. The resulting 243,488 probes spanned 844,339 bp (GRCh37/hg19), and included 71.1%, 72.2%, 88.4%, and 98.6% of the padded target, unpadded target, UCSC exons, and CCDS coding sequence, respectively. The probes and their reverse complements were each applied in duplicate to the capture array for a total of 973,952 probes.

For the second 1M SureSelect DNA capture array, Biomart (http://uswest.ensembl.org/biomart/) was used to obtain the Ensembl NCBI37/hg19 coordinates for the exons of 76 genes (Table S2). Exons were padded with 90 bp to define a 718,566 bp target region. eArray was used to design 568,634 probes to target the repeat masked sequences of this region (91%). The target region for *PTPN11* was defined as 93,180 bp spanning 1 kb upstream to 2 kb downstream of the gene. Repeat masker (http://www.repeatmasker.org/) was used to mask only Alu repeats (37% of the region) resulting in a target region of 61,365 bp, for which 55,205 probes were designed using eArray. All probes were 60-nt in length and spaced every 1-nt. The capture array was designed to include all probes (623,839 total), as well as the reverse complement of every 2^nd^ probe and every 17^th^ probe, for a total of 972,455 probes.

### Array hybridization

For the first capture array, 1.4 µg of each of the 16 amplified libraries was pooled and hybridized to the array following Agilent's SureSelect DNA Capture Array protocol version 1.0. Different blocking oligonucleotides (Table S5) were added to the hybridization. After elution from the array, half of the captured library was amplified in five 50 µl PCR reactions for 18 cycles using Phusion High-Fidelity DNA polymerase (Finnzymes) and post-capture primer pair (Table S5), purified using an E-Gel CloneWell (Invitrogen) to remove primer dimers, and re-amplified using fifteen PCR cycles with the same primer pair. The amplified library was again purified to eliminate primer dimers using E-Gel. For the second capture array, 2 µg of each of the 12 amplified libraries was pooled and hybridized to the array. After elution, half of the captured library was amplified in five 50 µl PCR reactions for 15 cycles and purified using Agencourt AMPure XP magnetic beads. Further details of the array design and methods for sequence analysis are provided in the supporting information. Additional methods for Illumina data analysis, copy number analysis, Sanger sequence analysis of *PTPN11* (Table S6), DNA extraction from lesional tissue, PCR product subcloning experiments, aCGH analysis, MLPA (Table S7), and immunodetection of SHP2 are also provided in the supporting information (Text S1).

## Supporting Information

Figure S1 Linkage mapping of metachondromatosis to chromosome 12q. A whole genome LOD score plot is depicted. The chromosomes are color-coded according to the key on the right. Raw genotype data were generated using Affymetrix 6.0 SNP arrays and multipoint parametric linkage analysis was performed using MERLIN with Affymetrix Caucasian allele frequencies and deCODE Genetics genetic map positions. The disease allele frequency was estimated at 1E-7, and phenocopies and non-penetrance were not permitted (affectation probability 0/1/1). Error checking identified and removed unlikely genotypes during initial analysis, and the cleaned data were then reanalyzed. Because non-penetrance and non-ascertainment are potential confounding factors in the diagnosis of MC, we analyzed only founders and affected individuals. Our analysis identified one interval on chromosome 12 (orange above), from 121.5 to 132.3 cM, that attained the maximum LOD score of 2.7. The minimum linked interval is bounded by rs1861693 (LOD score 2.1; GRCh37/hg19 coordinate 107603157) and rs1520173 (2.3; 116182525), and is flanked proximally by rs12422243 (−5.8; 107595567) and distally by rs2460488 (−4.3; 116187660), for a maximum linked interval of 8,592,092 bp. We did not observe any other autosomal interval >1 cM with a peak LOD score >−1.9. Several autosomal intervals <1 cM attained LOD scores >0, but we considered these unlikely to be candidate intervals and instead assumed they represented either unfiltered genotyping errors or short ancestral population haplotypes, rather than familial haplotypes inherited from a common ancestor. Although MC is well documented as autosomal dominant and many of our own pedigrees show male-to-male inheritance, family A does not; therefore we also examined the X chromosome. Four X-linked intervals >1 cM attained LOD scores >−2, but the largest was 3.4 cM and the highest LOD score was 0.3; we therefore assumed these intervals also represented either unfiltered genotyping errors or short ancestral population haplotypes.
(PDF)

Figure S2 Amplified libraries before and after array capture. (A) Bioanalyzer plot of a representative amplified genomic DNA library before targeted array capture. Peaks at 15 bp and 1500 bp represent size standards. The broad peak between 100 and 300 bp comprises the barcoded and amplified DNA library. (B) Photograph of an Ethidium Bromide stained 4% agarose gel containing the pooled library after targeted array capture and amplification, but before E-gel purification. Note the broad band of amplimer between 200 bp and 300 bp in size. These sized amplimers were used to obtain Illumina GA II sequence.
(PDF)

Figure S3 Specificity of target capture and read depth of targeted bases. (A) Graph depicting the number of filtered Illumina GA II reads per barcoded sample that aligned to the targeted region (dark blue), aligned elsewhere in the human genome (medium blue), or failed to align to the reference genome (light blue), for both the first (left) and second (right) capture array experiment. Each letter refers to a family and if more than one member of the family was sequenced then the specific family member is indicated. For each array, of the 50 million reads obtained from two lanes of GA II sequencing, between 1 and 6 million reads were obtained for each individual. The percentage of each individual's reads that mapped to the targted region was ∼60% for the first array and ∼40% for the second array. (B) Percentage of bases targeted by the array having read depths greater than or equal to a specific fold-coverage. Graph depicting the mean of the percent of targeted bases captured in each individual at greater than or equal to 1, 5, 10, 20, 30, 40, and 50-fold coverage (nX). Values are shown before (dark red) and after (light red) removal of PCR duplicates. Error bars indicate 1 SD.
(PDF)

Figure S4 Identified coding variants in unrelated individuals from the first capture array. Total coding variants identified in the 8 unrelated individuals who were the only members of their families included in the first array capture experiment. The graph depicts the number of variants and the number of reads containing each variant in an individual. Novel (dark gray) and previously characterized (light gray) SNPs are separated into different bars. Note that the ratio between novel and known SNPs decreases as the number of reads containing that SNP increases. These data suggest that most novel SNPs seen in 3 or fewer reads represent sequence artifacts rather than real variants. Shown above the graph are novel variants that were observed in three or more reads in single individuals, with the gene names and predicted amino acid changes indicated. Nonsense mutations are indicated in red, nonsynonomous mutations are indicated in purple, and synonomous mutations are indicated in green. No insertion or deletion variants were seen in more than 2 reads. The family in which the novel variant was identified is indicated in parentheses. Two mutations were identified in *PTPN11*, including a nonsense mutation and a synonomous mutation. The nonsense mutation was confirmed by Sanger sequence analysis; however, the synonomous mutation could not be confirmed and therefore likely represents a false positive result.
(PDF)

Figure S5 Heterozygous deletions of *EXT1* identified in two participants. (A) Log2 values of the number of Illumina reads obtained per 50 bp window in participants N and Q, divided by the average number of reads obtained in other participants whose DNA was captured simultaneously using the second capture array. Shown are all 50 bp windows spanning the exons of four genes (*FGFR1, LYN, EXT1, PTK2*) located on chromosome 8 that were targeted by the capture array. The red bars indicate a region spanning all *EXT1* exons in participant N, and exon 1 of *EXT1* in participant Q, where consecutive windows have log 2 values of approximately −1, suggesting heterozygous deletions of these regions. (B) MLPA amplification products separated by electrophoresis. The amplification products of the MLPA probes targeting both the middle and the boundary of *EXT1* exon 1, and the MLPA probes targeting the middle of *EXT1* exon 1, have reduced peak heights in participants N and Q respectively (arrowheads). (C) Ratio of each *EXT1* peak height to the average height of the four control peaks. The ratios were normalized based on the ratios obtained in control individuals, such that a ratio of 1.0 indicates a copy number of 2, while a ratio less than the threshold of 0.8 suggests a copy number of 1. In both participants N and Q, the ratio for the probe corresponding to the middle of *EXT1* exon 1 is below 0.8, suggesting a heterozygous deletion of this exon. MLPA was performed twice for each participant. Error bars indicate standard deviation.
(PDF)

Figure S6 Truncated SHP2 protein is not detected. Immunoblots of protein extracted from an exostosis from participant B-IV-7 with a truncating frameshift mutation, rib cartilage from an unaffected individual (cartilage expression tissue control), and HELA cells (antibody control) that have been separated on a 4–12% SDS-PAGE gel, transferred to Immobilon-P. Upper panel: immunodetection using mouse-anti-SHP2 that detects an epitope N-terminal of the frameshift mutation. Brackets indicate wild-type SHP2 that is differentially phosphorylated. Wild-type protein is present in the exostosis, likely from the bone marrow and fibrous elements since the lesional cartilage was not selectively isolated. Note absence of a unique band 17 kD in size, that would have been expected if truncated protein was produced (arrow). Lower panel: immunodetection of the same blot using a mouse-anti-actin antibody as a loading control.
(PDF)

Table S1 Summary of metachondromatosis families and *PTPN11* mutations.
(DOC)

Table S2 Genes included in second capture array.
(DOC)

Table S3 Mutations identified in second capture array.
(DOC)

Table S4 Barcoded library adapters.
(DOC)

Table S5 PCR primers for library amplification and blocking oligonucleotides.
(DOC)

Table S6 Primers used for amplification of *PTPN11* coding exons.
(DOC)

Table S7 Probes used for MLPA.
(DOC)

Text S1 Supporting Materials and Methods.
(DOC)
