# Supplementary figures and images for: Loss-of-Function Mutations in PTPN11 Cause Metachondromatosis, but Not Ollier Disease or Maffucci Syndrome
Source: PLoS Genet. 2011 Apr 14;7(4):e1002050. doi: 10.1371/journal.pgen.1002050 (PMC3077396; doi:10.1371/journal.pgen.1002050)

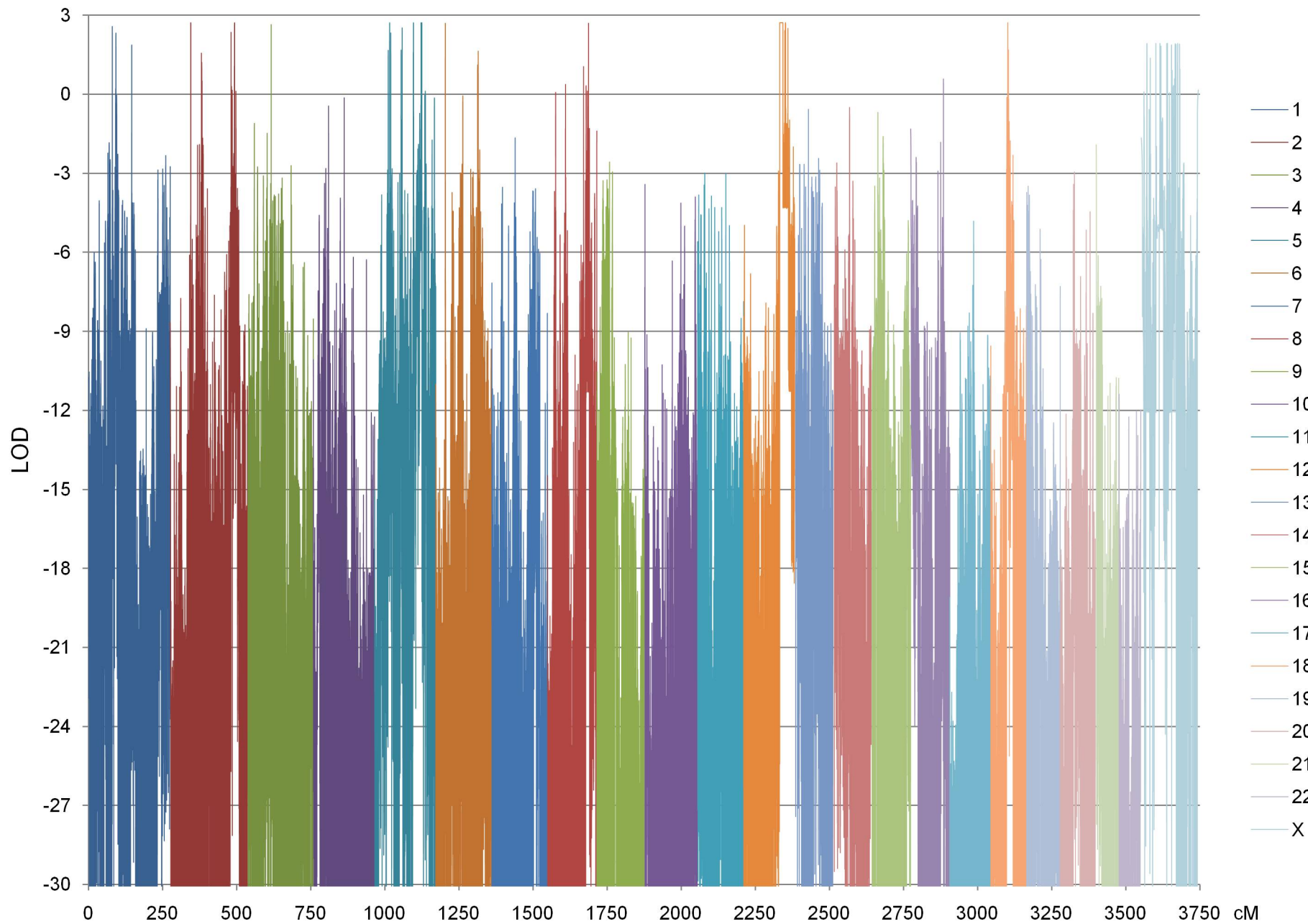

Supplement: Figure S1 — Linkage mapping of metachondromatosis to chromosome 12q. A whole genome LOD score plot is depicted. The chromosomes are color-coded according to the key on the right. Raw genotype data were generated using Affymetrix 6.0 SNP arrays and multipoint parametric linkage analysis was performed using MERLIN with Affymetrix Caucasian allele frequencies and deCODE Genetics genetic map positions. The disease allele frequency was estimated at 1E-7, and phenocopies and non-penetrance were not permitted (affectation probability 0/1/1). Error checking identified and removed unlikely genotypes during initial analysis, and the cleaned data were then reanalyzed. Because non-penetrance and non-ascertainment are potential confounding factors in the diagnosis of MC, we analyzed only founders and affected individuals. Our analysis identified one interval on chromosome 12 (orange above), from 121.5 to 132.3 cM, that attained the maximum LOD score of 2.7. The minimum linked interval is bounded by rs1861693 (LOD score 2.1; GRCh37/hg19 coordinate 107603157) and rs1520173 (2.3; 116182525), and is flanked proximally by rs12422243 (−5.8; 107595567) and distally by rs2460488 (−4.3; 116187660), for a maximum linked interval of 8,592,092 bp. We did not observe any other autosomal interval >1 cM with a peak LOD score >−1.9. Several autosomal intervals <1 cM attained LOD scores >0, but we considered these unlikely to be candidate intervals and instead assumed they represented either unfiltered genotyping errors or short ancestral population haplotypes, rather than familial haplotypes inherited from a common ancestor. Although MC is well documented as autosomal dominant and many of our own pedigrees show male-to-male inheritance, family A does not; therefore we also examined the X chromosome. Four X-linked intervals >1 cM attained LOD scores >−2, but the largest was 3.4 cM and the highest LOD score was 0.3; we therefore assumed these intervals also represented either unfiltered genotyping errors or [file pgen.1002050.s001.pdf]

**A**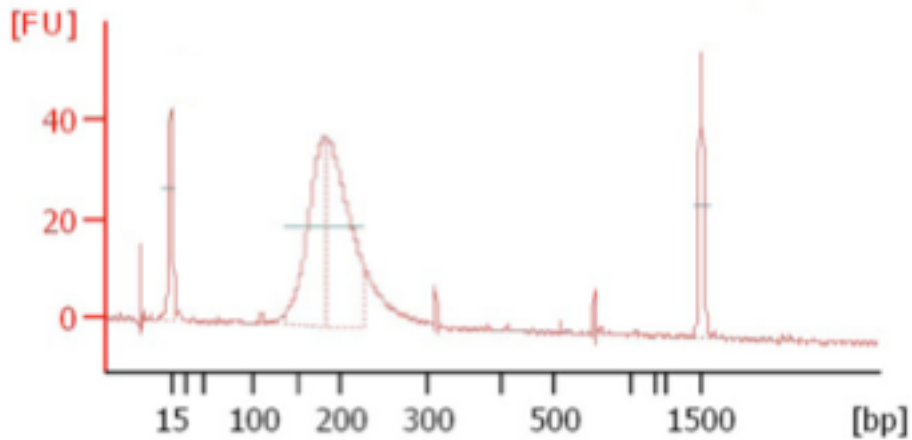**B**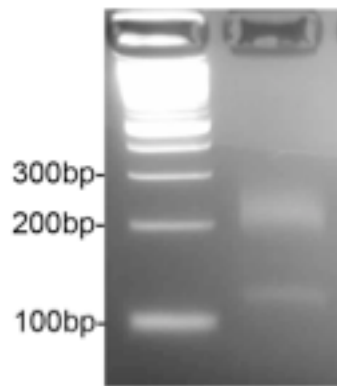

Supplement: Figure S2 — Amplified libraries before and after array capture. (A) Bioanalyzer plot of a representative amplified genomic DNA library before targeted array capture. Peaks at 15 bp and 1500 bp represent size standards. The broad peak between 100 and 300 bp comprises the barcoded and amplified DNA library. (B) Photograph of an Ethidium Bromide stained 4% agarose gel containing the pooled library after targeted array capture and amplification, but before E-gel purification. Note the broad band of amplimer between 200 bp and 300 bp in size. These sized amplimers were used to obtain Illumina GA II sequence. (PDF) [file pgen.1002050.s002.pdf]

**A**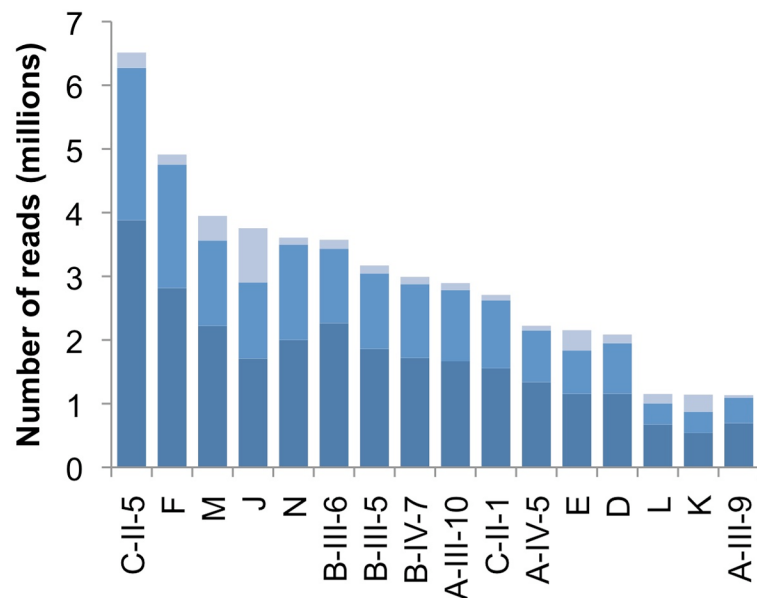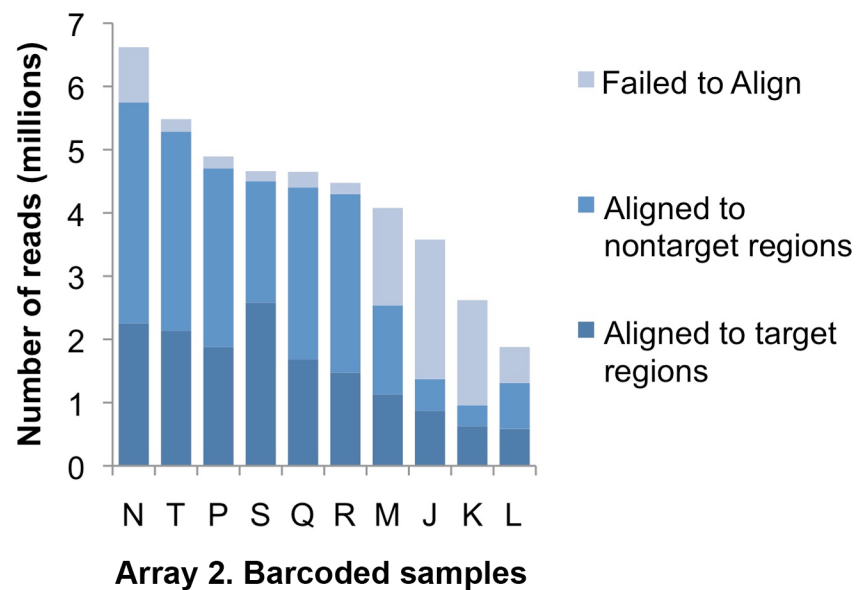**B**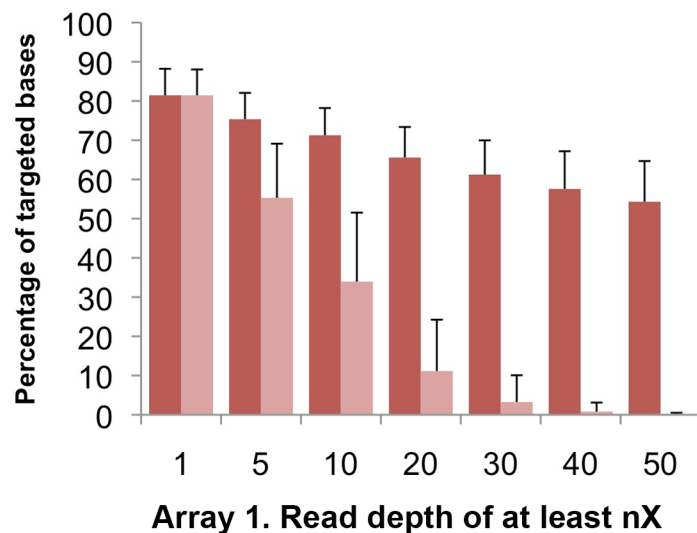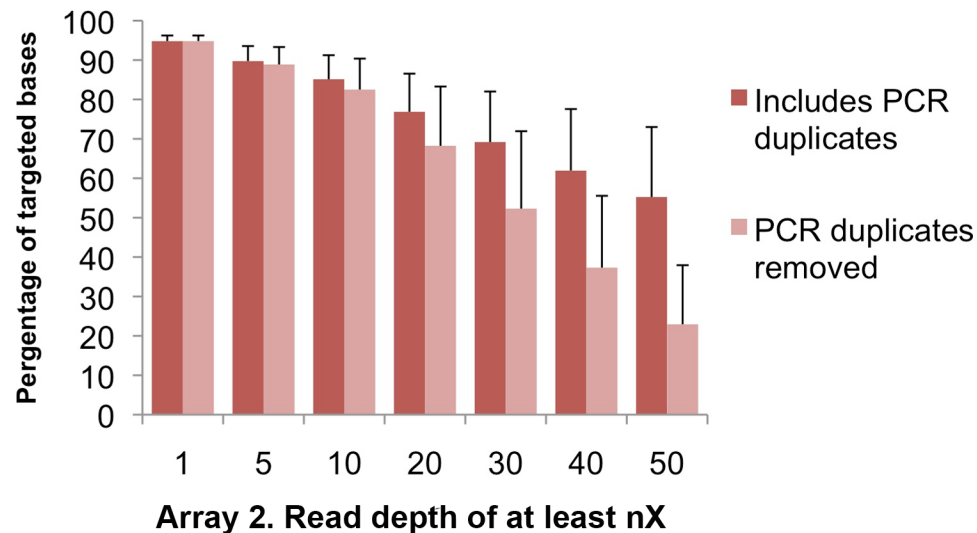

Supplement: Figure S3 — Specificity of target capture and read depth of targeted bases. (A) Graph depicting the number of filtered Illumina GA II reads per barcoded sample that aligned to the targeted region (dark blue), aligned elsewhere in the human genome (medium blue), or failed to align to the reference genome (light blue), for both the first (left) and second (right) capture array experiment. Each letter refers to a family and if more than one member of the family was sequenced then the specific family member is indicated. For each array, of the 50 million reads obtained from two lanes of GA II sequencing, between 1 and 6 million reads were obtained for each individual. The percentage of each individual's reads that mapped to the targted region was ∼60% for the first array and ∼40% for the second array. (B) Percentage of bases targeted by the array having read depths greater than or equal to a specific fold-coverage. Graph depicting the mean of the percent of targeted bases captured in each individual at greater than or equal to 1, 5, 10, 20, 30, 40, and 50-fold coverage (nX). Values are shown before (dark red) and after (light red) removal of PCR duplicates. Error bars indicate 1 SD. (PDF) [file pgen.1002050.s003.pdf]

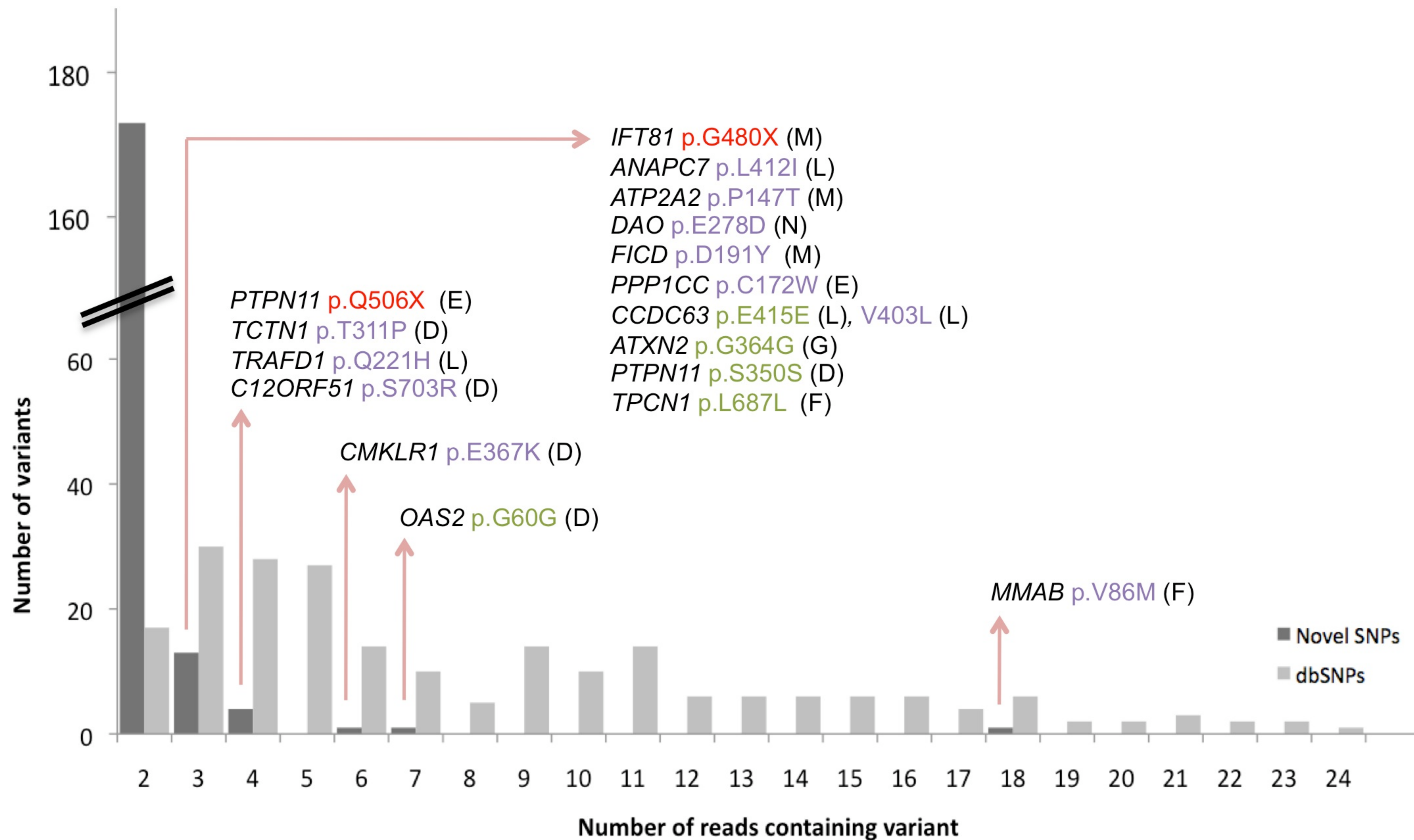

Supplement: Figure S4 — Identified coding variants in unrelated individuals from the first capture array. Total coding variants identified in the 8 unrelated individuals who were the only members of their families included in the first array capture experiment. The graph depicts the number of variants and the number of reads containing each variant in an individual. Novel (dark gray) and previously characterized (light gray) SNPs are separated into different bars. Note that the ratio between novel and known SNPs decreases as the number of reads containing that SNP increases. These data suggest that most novel SNPs seen in 3 or fewer reads represent sequence artifacts rather than real variants. Shown above the graph are novel variants that were observed in three or more reads in single individuals, with the gene names and predicted amino acid changes indicated. Nonsense mutations are indicated in red, nonsynonomous mutations are indicated in purple, and synonomous mutations are indicated in green. No insertion or deletion variants were seen in more than 2 reads. The family in which the novel variant was identified is indicated in parentheses. Two mutations were identified in PTPN11, including a nonsense mutation and a synonomous mutation. The nonsense mutation was confirmed by Sanger sequence analysis; however, the synonomous mutation could not be confirmed and therefore likely represents a false positive result. (PDF) [file pgen.1002050.s004.pdf]

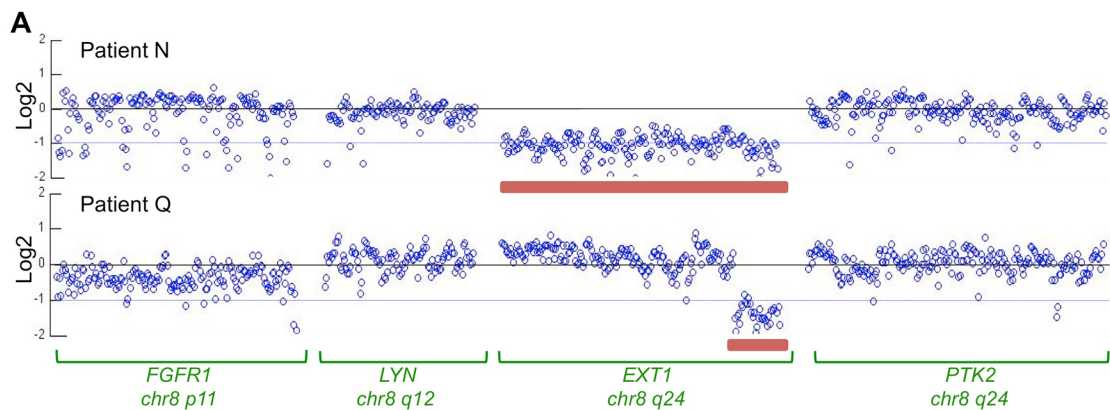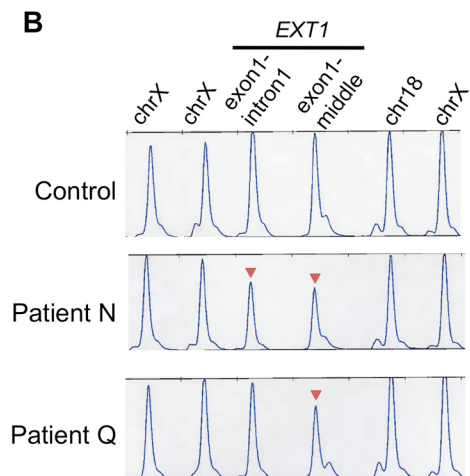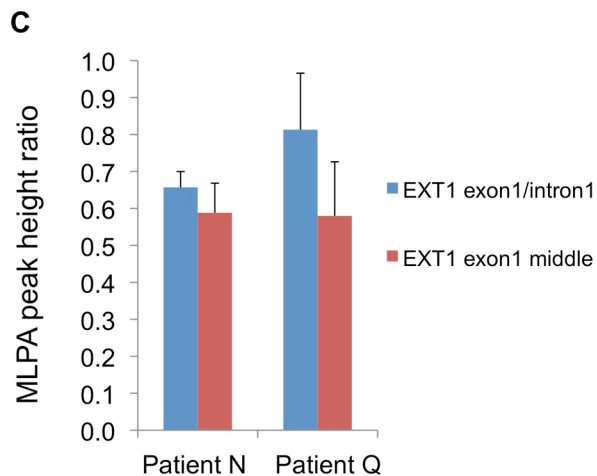

Supplement: Figure S5 — Heterozygous deletions of EXT1 identified in two participants. (A) Log2 values of the number of Illumina reads obtained per 50 bp window in participants N and Q, divided by the average number of reads obtained in other participants whose DNA was captured simultaneously using the second capture array. Shown are all 50 bp windows spanning the exons of four genes (FGFR1, LYN, EXT1, PTK2) located on chromosome 8 that were targeted by the capture array. The red bars indicate a region spanning all EXT1 exons in participant N, and exon 1 of EXT1 in participant Q, where consecutive windows have log 2 values of approximately −1, suggesting heterozygous deletions of these regions. (B) MLPA amplification products separated by electrophoresis. The amplification products of the MLPA probes targeting both the middle and the boundary of EXT1 exon 1, and the MLPA probes targeting the middle of EXT1 exon 1, have reduced peak heights in participants N and Q respectively (arrowheads). (C) Ratio of each EXT1 peak height to the average height of the four control peaks. The ratios were normalized based on the ratios obtained in control individuals, such that a ratio of 1.0 indicates a copy number of 2, while a ratio less than the threshold of 0.8 suggests a copy number of 1. In both participants N and Q, the ratio for the probe corresponding to the middle of EXT1 exon 1 is below 0.8, suggesting a heterozygous deletion of this exon. MLPA was performed twice for each participant. Error bars indicate standard deviation. (PDF) [file pgen.1002050.s005.pdf]

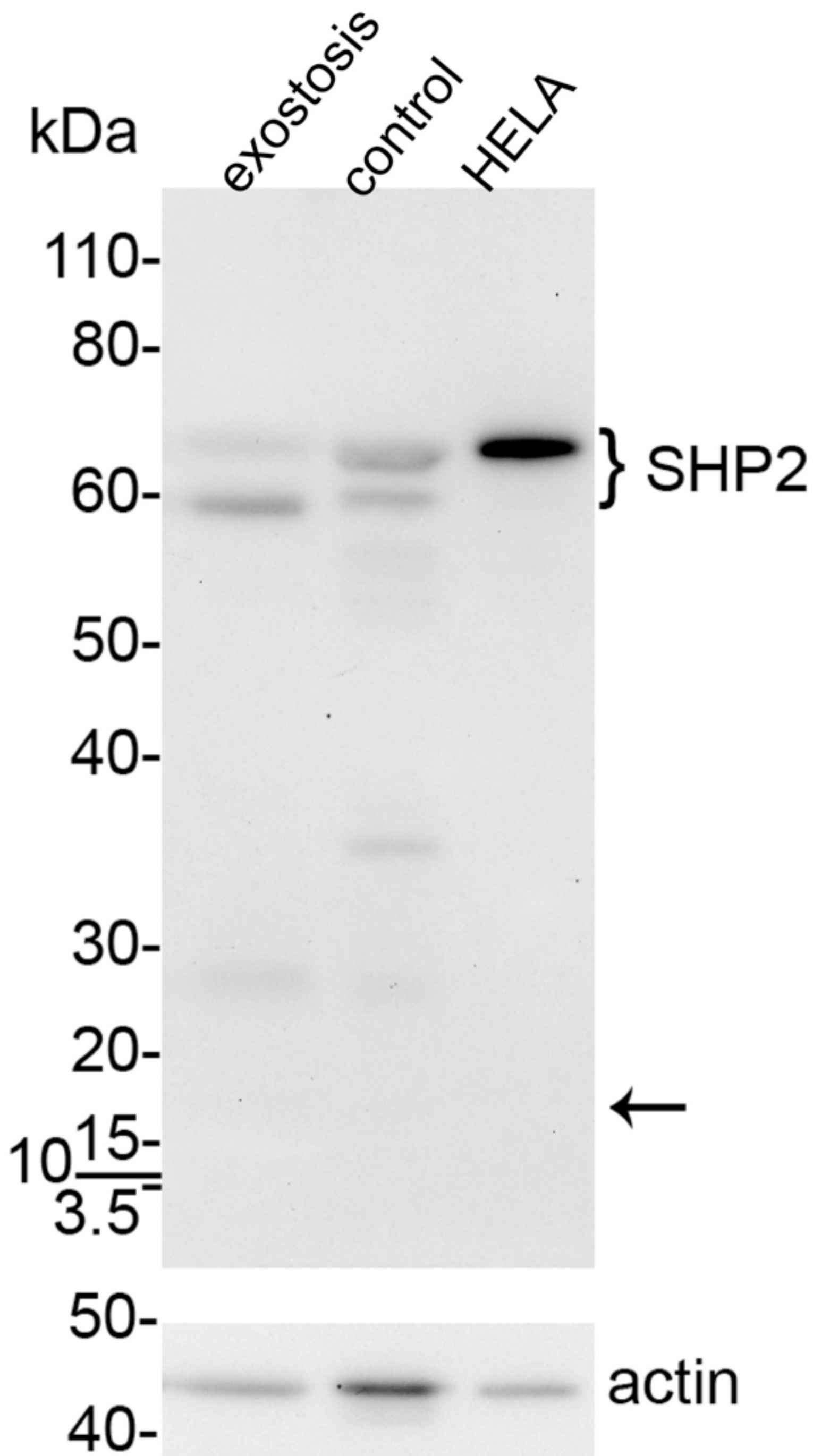

Supplement: Figure S6 — Truncated SHP2 protein is not detected. Immunoblots of protein extracted from an exostosis from participant B-IV-7 with a truncating frameshift mutation, rib cartilage from an unaffected individual (cartilage expression tissue control), and HELA cells (antibody control) that have been separated on a 4–12% SDS-PAGE gel, transferred to Immobilon-P. Upper panel: immunodetection using mouse-anti-SHP2 that detects an epitope N-terminal of the frameshift mutation. Brackets indicate wild-type SHP2 that is differentially phosphorylated. Wild-type protein is present in the exostosis, likely from the bone marrow and fibrous elements since the lesional cartilage was not selectively isolated. Note absence of a unique band 17 kD in size, that would have been expected if truncated protein was produced (arrow). Lower panel: immunodetection of the same blot using a mouse-anti-actin antibody as a loading control. (PDF) [file pgen.1002050.s006.pdf]
