# Supplementary material for: Loss-of-Function Mutations in PTPN11 Cause Metachondromatosis, but Not Ollier Disease or Maffucci Syndrome
Source: PLoS Genet. 2011 Apr 14;7(4):e1002050. doi: 10.1371/journal.pgen.1002050 (PMC3077396; doi:10.1371/journal.pgen.1002050)
Supplement: Table S1 — Summary of metachondromatosis families and PTPN11 mutations. (DOC) [file pgen.1002050.s007.doc]

**Table S1. Summary of metachondromatosis families and *PTPN11* mutations**

|  | **Family** | **Familial/ sporadic** | **# Affecteds collected** | **Referral site** | **DNA change (chr12, hg19 build)** | **cDNA change** | **Predicted protein change** | **Mutation type** | **Mutation site** | **Method Identified** | **Included in Next Gen^1^** | **Included in aCGH^1^** |
| --- | --- | --- | --- | --- | --- | --- | --- | --- | --- | --- | --- | --- |
| 1 | A | F | 10 | USA | g.112,891,075 del5 | c.409_413del5 | p.V137RfsX17 | frameshift | exon 4 | Next Gen 1 | 2A, 1U (III-9,10, IV-5) | 2A |
| 2 | B | F | 2 | USA | g.112,891,124 del11ins24^2^ | c.458_468 del11ins24 | p.T153KfsX8 | frameshift | exon 4 | Next Gen 1 | 2A, 1U (III-5, 6, IV-7) | 1A |
| 3 | C | F | 4 | Switzerland | g.112,891,019 del2 | c.353_354del2 | p.S118WfsX10 | frameshift | exon 4 | Next Gen 1 | 2A (II-5, III-1) | 1A |
| 4 | I | F | 2 | UK | g.112,888,279 A>T | c.295A>T | p.K99X | nonsense | exon 3 | Sanger |  |  |
| 5 | D | F | 2 | Japan | g.112,924,369 del1 | c.1315del1 | p.L439WfsX33 | frameshift | exon 11 | Sanger | 1A | 1A |
| 6 | E^3^ | F | 3 | Japan | g.112,926,896 C>T | c.1516C>T | p.Q506X | nonsense | exon 13 | Next Gen 1 | 1A | 1A |
| 7 | J | S | 1 | Japan |  |  |  |  |  |  | 1A |  |
| 8 | K | S | 1 | Japan |  |  |  |  |  |  | 1A |  |
| 9 | L | S | 1 | Australia |  |  |  |  |  |  | 1A | 1A |
| 10 | M | S | 1 | Australia |  |  |  |  |  |  | 1A |  |
| 11 | F | F | 3 | France | g.112,893,752 A>C | c.643-2A>C | unknown | splice site | intron 5 | Sanger | 1A |  |
| 12 | G | F | 1 | France | g.112,919,877 G>T | c.1093-1G>T | unknown | splice site | intron 9 | Sanger |  |  |
| 13 | S | S | 1 | Italy | g.112,897,487del14629ins2 |  | p.T253LfsX54 | frameshift | exon 7 | Next Gen 2 | 1A |  |
| 14 | T | S | 1 | Italy |  |  |  |  |  |  |  |  |
| 15 | H | F | 1 | Belgium | g.112,893,791 del4 | c.680_683del4 | p.E227AfsX6 | frameshift | exon 10 | Sanger |  |  |
| 16 | O^4^ | S | 1 | Netherlands | g.112,924,278G>A | c.1225-1G>A | unknown | splice site | intron 10 | Sanger |  |  |
| 17 | P^4^ | F | 1 | Netherlands |  |  |  |  |  |  |  |  |

^1^A= Affected, U= Unaffected.

^2^Insertion of the following 24 bp sequence: AAGAACACAGGGGAGAGCAATGAC.

^3^Patients previously published in Reference 8.

^4^Patients previously published in Reference 10.
