# Supplementary material for: Loss-of-Function Mutations in PTPN11 Cause Metachondromatosis, but Not Ollier Disease or Maffucci Syndrome
Source: PLoS Genet. 2011 Apr 14;7(4):e1002050. doi: 10.1371/journal.pgen.1002050 (PMC3077396; doi:10.1371/journal.pgen.1002050)
Supplement: Table S2 — Genes included in second capture array. (DOC) [file pgen.1002050.s008.doc]

**Table S2. Genes included in second capture array**

| **Category** | **Gene** |
| --- | --- |
| Ras/MAPK pathway | *KRAS, HRAS, NRAS, ARAF, BRAF, RAF1, MAPK1, MAPK3, MAP2K1, MAP2K2, SOS1, SOS2, GRB2, SHOC2, GAB1, GAB2, GAB3, FRS2, IRS1, SHC1, SIRPA, PXN, MVP, MPZL1, CRK* |
| PI3K/AKT pathway | *AKT1, AKT2, AKT3, PIK3R1, PIK3R2, PIK3R3, PIK3CA, PIK3CB, PIK3C2A, PIK3C2B, PIK3C2G, MTOR, RPS6KB1, PDPK1, ILK, PRKCD, PRKCZ, PTEN* |
| Receptor Tyrosine Kinases | *FGFR1, FGFR2, FGFR3, EGFR, PDGFRA, PDGFRB, IGF1R, ERBB2, ERBB3, ERBB4, ROR1, ROR2, MUSK, AXL, MERTK, DDR1, DDR2* |
| Other | *EXT1, EXT2, RHOA, CDC42, RAC1, PTH1R, SRC, PTK2, YES1, FYN, LYN, FGR, PLCG1, PLCG2, HSPA4, INPP5D* |
