# Supplementary material for: Loss-of-Function Mutations in PTPN11 Cause Metachondromatosis, but Not Ollier Disease or Maffucci Syndrome
Source: PLoS Genet. 2011 Apr 14;7(4):e1002050. doi: 10.1371/journal.pgen.1002050 (PMC3077396; doi:10.1371/journal.pgen.1002050)
Supplement: Table S3 — Mutations identified in second capture array. (DOC) [file pgen.1002050.s009.doc]

**Table S3. Mutations identified in second capture array**

| **Location** | **Patient** | **Mutation** |
| --- | --- | --- |
| ***PTPN11*** | | **Chr12 genomic change** |
| **3’ UTR** | |  |
|  | M | g.112945402G>A |
| **Intronic** | | |
|  | J | g.112857243G>T, g.112869736del1, g.112924937C>T |
|  | M | g.112862223C>T, g.112914741C>G, g.112933866A>G, g.112945402G>A |
|  |  |  |
| **Coding mutations** | | **Gene name and predicted protein change** |
|  | J | *MTOR* p.P1408S, *MVP* p.R49S |
|  | K | *ERBB2* p.S1050L, *MAP2K2* p.D140D |
|  | L | *MVP* p.T199T |
|  | M | *PIK3C2B* p.D478D |
|  | P | *SOS2* p.D952N, *RAF1* p.L351L |
