# Supplementary material for: Loss-of-Function Mutations in PTPN11 Cause Metachondromatosis, but Not Ollier Disease or Maffucci Syndrome
Source: PLoS Genet. 2011 Apr 14;7(4):e1002050. doi: 10.1371/journal.pgen.1002050 (PMC3077396; doi:10.1371/journal.pgen.1002050)
Supplement: Table S4 — Barcoded library adapters. (DOC) [file pgen.1002050.s010.doc]

**Table S4. Barcoded library adapters**

| **AAC** | 5’-ACACTCTTTCCCTACACGACGCTCTTCCGATCT**AAC***T-3’ |
| --- | --- |
|  | 5’-/5Phos/**GTT**AGATCGGAAGAGCGGTTCAGCAGGAATGCCGAG-3’ |
| **AGC** | 5’-ACACTCTTTCCCTACACGACGCTCTTCCGATCT**AGC***T-3’ |
|  | 5’-/5Phos/**GCT**AGATCGGAAGAGCGGTTCAGCAGGAATGCCGAG-3’ |
| **AGG** | 5’-ACACTCTTTCCCTACACGACGCTCTTCCGATCT**AGG***T-3’ |
|  | 5’-/5Phos/**CCT**AGATCGGAAGAGCGGTTCAGCAGGAATGCCGAG-3’ |
| **CAG** | 5’-ACACTCTTTCCCTACACGACGCTCTTCCGATCT**CAG***T-3’ |
|  | 5’-/5Phos/**CTG**AGATCGGAAGAGCGGTTCAGCAGGAATGCCGAG-3’ |
| **CGA** | 5’-ACACTCTTTCCCTACACGACGCTCTTCCGATCT**CGA***T-3’ |
|  | 5’-/5Phos/**TCG**AGATCGGAAGAGCGGTTCAGCAGGAATGCCGAG-3’ |
| **CGT** | 5’-ACACTCTTTCCCTACACGACGCTCTTCCGATCT**CGT***T-3’ |
|  | 5’-/5Phos/**ACG**AGATCGGAAGAGCGGTTCAGCAGGAATGCCGAG-3’ |
| **CTC** | 5’-ACACTCTTTCCCTACACGACGCTCTTCCGATCT**CTC***T-3’ |
|  | 5’-/5Phos/**GAG**AGATCGGAAGAGCGGTTCAGCAGGAATGCCGAG-3’ |
| **GAC** | 5’-ACACTCTTTCCCTACACGACGCTCTTCCGATCT**GAC***T-3’ |
|  | 5’-/5Phos/**GTC**AGATCGGAAGAGCGGTTCAGCAGGAATGCCGAG-3’ |
| **GCA** | 5’-ACACTCTTTCCCTACACGACGCTCTTCCGATCT**GCA***T-3’ |
|  | 5’-/5Phos/**TGC**AGATCGGAAGAGCGGTTCAGCAGGAATGCCGAG-3’ |
| **GGA** | 5’-ACACTCTTTCCCTACACGACGCTCTTCCGATCT**GGA***T-3’ |
|  | 5’-/5Phos/**TCC**AGATCGGAAGAGCGGTTCAGCAGGAATGCCGAG-3’ |
| **GTC** | 5’-ACACTCTTTCCCTACACGACGCTCTTCCGATCT**GTC***T-3’ |
|  | 5’-/5Phos/**GAC**AGATCGGAAGAGCGGTTCAGCAGGAATGCCGAG-3’ |
| **GTG** | 5’-ACACTCTTTCCCTACACGACGCTCTTCCGATCT**GTG***T-3’ |
|  | 5’-/5Phos/**CAC**AGATCGGAAGAGCGGTTCAGCAGGAATGCCGAG-3’ |
| **TCC** | 5’-ACACTCTTTCCCTACACGACGCTCTTCCGATCT**TCC***T-3’ |
|  | 5’-/5Phos/**GGA**AGATCGGAAGAGCGGTTCAGCAGGAATGCCGAG-3’ |
| **TCG** | 5’-ACACTCTTTCCCTACACGACGCTCTTCCGATCT**TCG***T-3’ |
|  | 5’-/5Phos/**CGA**AGATCGGAAGAGCGGTTCAGCAGGAATGCCGAG-3’ |
| **TGA** | 5’-ACACTCTTTCCCTACACGACGCTCTTCCGATCT**TGA***T-3’ |
|  | 5’-/5Phos/**TCA**AGATCGGAAGAGCGGTTCAGCAGGAATGCCGAG-3’ |
| **TGG** | 5’-ACACTCTTTCCCTACACGACGCTCTTCCGATCT**TGG***T-3’ |
|  | 5’-/5Phos/**CCA**AGATCGGAAGAGCGGTTCAGCAGGAATGCCGAG-3’ |

Asterisk (*) indicates a phosphorothioate bond to prevent degradation.

Adapter sequences are Illumina paired end adapters with a 3bp identifying barcode indicated in bold type.

Library adapters © 2006 Illumina, Inc. All rights reserved.
