# Supplementary material for: Loss-of-Function Mutations in PTPN11 Cause Metachondromatosis, but Not Ollier Disease or Maffucci Syndrome
Source: PLoS Genet. 2011 Apr 14;7(4):e1002050. doi: 10.1371/journal.pgen.1002050 (PMC3077396; doi:10.1371/journal.pgen.1002050)
Supplement: Table S5 — PCR primers for library amplification and blocking oligonucleotides. (DOC) [file pgen.1002050.s011.doc]

**Table S5. PCR primers for library amplification and blocking oligonucleotides**

| **Pre-capture Primers** |  |
| --- | --- |
| Pre-capture Fw | 5’-ACACTCTTTCCCTACACGACGCT-3’ |
| Pre-capture Rev | 5’-GGTCTCGGCATTCCTGCTGAA-3’ |
|  |  |
| **Post-capture Primers** |  |
| Post-capture Fw^1^ | 5'- AATGATACGGCGACCACCGAGATCTACACTCTTTCCCTACACGACGCTCTTCCGATC*T-3’ |
| Post-capture Rev^2^ | 5'- CAAGCAGAAGACGGCATACGAGATCGGTCTCGGCATTCCTGCTGAACCGCTCTTCCGATC* T-3’ |
|  |  |
| **Blocking Oligos** |  |
| BO_A (Adapter sequence 1) | 5'-ACACTCTTTCCCTACACGACGCTCTTCCGATCT-3' |
| Rev. comp. BO_A | 5'-AGATCGGAAGAGCGTCGTGTAGGGAAAGAGTGT-3' |
| BO_B (Adapter sequence 2) | 5'-GGTCTCGGCATTCCTGCTGAACCGCTCTTCCGATCT-3' |
| Rev. comp. BO_B | 5'-AGATCGGAAGAGCGGTTCAGCAGGAATGCCGAGACC-3' |

^1^ PCR primer © 2006 Illumina, Inc. All rights reserved.

^2^ Bentley *et al.* (2008) Nature Nov 6;456(7218):53-9
