# Supplementary material for: Loss-of-Function Mutations in PTPN11 Cause Metachondromatosis, but Not Ollier Disease or Maffucci Syndrome
Source: PLoS Genet. 2011 Apr 14;7(4):e1002050. doi: 10.1371/journal.pgen.1002050 (PMC3077396; doi:10.1371/journal.pgen.1002050)
Supplement: Table S6 — Primers used for amplification of PTPN11 coding exons. (DOC) [file pgen.1002050.s012.doc]

**Table S6. Primers used for amplification of *PTPN11* coding exons**

| **Exon** | **Forward primer** | | **Reverse primer** | | **PCR product size (bp)** |
| --- | --- | --- | --- | --- | --- |
|  | **Sequence^1^** | **Position^2^** | **Sequence^3^** | **Position^2^** |  |
| 1 | GGGCAGCTGCACAGTCTC | -178 to -160 | GGGGACGAGGAGGGAAC | +143 to +127 | 334 |
| 1S^4^ | CGGAGCCTGAGCAAGGAG | -54 to -35 | CTTCCGGACGGGGCTAAC | +71 to +56 | 139 |
| 2 | AAGGGACAGGGAAGGTCTTG | -137 to -118 | AACCCTGAAGGCAGCCC | +133 to +117 | 393 |
| 2S^4^ | AGTGCTGACAGTGTCTTGTTTTT | -52 to -30 | CTCTCAGGATCCTCTCTTTTCA | +33 to +12 | 208 |
| 3 | TGTGGTTATTTCACCCATCG | -163 to -144 | AAAGGTACTCTGAAAATAATTTGATGC | +100 to +121 | 539 |
| 3S^4^ | AACTCTTTATTTGTCCCCTTGC | -47 to -26 | AGGGAGCAGCAGACTTTGTG | +40 to +21 | 284 |
| 4 | TGATCAATCCCTTGGAGGAA | -134 to -115 | GAAAAATCACCCAAAGGTAACA | +56 to +35 | 383 |
| 4B | TGAAAGAACAACATGAACCCATA | -61 to -38 | GTGCGCACAGAAAGAACAAA | -68 to -87 | 187 |
| 4S1^4^ | TGAAAGAACAACATGAACCCATA | -61 to -39 | GTGCGCACAGAAAGAACAAA | c.451 to c.471 | 187 |
| 4S2^4^ | CTTGTACGAGAGAGCCAGAGC | c.406 to c.427 | TCTTGCCAGACCCATTTTTC | +35 to +15 | 154 |
| 5 | CTGCAGTGAACATGAGAGTGC | -83 to -63 | TTCTGTCTACCTCTGTATGTTTGC | +89 to +66 | 289 |
| 5S^4^ | TGAGAGTGCTTGAAAACACTAATGTAA | -71 to -45 | TTTAAGTTTTTAAGGAGAAAGCTTGAG | +44 to +18 | 232 |
| 6 | TTTGCATTAACACCGTTTTCTG | -64 to -43 | CAAGAGCAACTTCTCCTCCA | +71 to +52 | 249 |
| 6S^4^ | CATCAACTGCTGTACTCGATCA | -23 to -6 | CAGGTCCAATTCCAAACACA | +89 to +70 | 226 |
| 7 | CCCAGATGAACATTCTTGTAGC | -145 to -126 | CAATTAAAGAAGCTAAGGCCTCC | +124 to +102 | 366 |
| 7S^4^ | TTTTTCTGTGACTCTTTGACACG | -52 to -30 | GGTGCTAGGAATCAAAATCTCC | +38 to +59 | 208 |
| 8&9 | TGGACTAGGCTGGGGAGTAA | -87 to -68 | TTCCTAAACATGGCCAATCTG | +55 to +76 | 527 |
| 8S^4^ | TGGACTAGGCTGGGGAGTAA | -68 to -87 | TTTCAGGACATGAGGAAGGA | +83 to +64 | 250 |
| 9S^4^ | TCCTTCCTCATGTCCTGAAAG | -63 to -43 | TTCCTAAACATGGCCAATCTG | +75 to +55 | 297 |
| 10 | CTAACAGATGCGAAACAGGC | -133 to -114 | GGAAGCTAATAAATGGCTACTGAATC | +137 to +112 | 402 |
| 10S^4^ | CAGAGTTCACAGAATTAACTTTCTTTT | -44 to -18 | TCCCAAAGTCTCTAGAATACGACA | +34 to +11 | 210 |
| 11 | GGACCTTTCAGTGGAACCTG | -128 to -109 | CAATGCAGTTGCTCTATGCC | +139 to +120 | 423 |
| 11S^4^ | CGGGTGATTCCTCAACCTCT | -64 to -45 | CGCTAGGAGACAGGGACAGG | +52 to +33 | 271 |
| 12 | CTGACTCCAAAGCCCTATGC | -173 to -154 | TCTGTTCTCAGGCAGACACAC | +154 to +134 | 395 |
| 12S^4^ | GAGTCTGAAACCCCCATGAA | -59 to -40 | CCAGACTGTTTTCGTGAGCA | +79 to +59 | 205 |
| 13 | CTGTAGCCATTGCAACATGC | -140 to -121 | TGGGAGTTTGTTTCCCATTC | +130 to +111 | 422 |
| 13S^4^ | CCTGGCTCTGCAGTTTCTCT | -47 to -28 | AGAGGCCTAGCAAGAGAATGA | +51 to +31 | 250 |
| 14 | TTTTTGCTTTTTATCCCCTTAAA | -64 to -43 | AAACCACCATGACCAGGTAAA | +46 to +26 | 223 |
| 15 | CAGGAACTGTGTCTGTACCATATC | -121 to -102 | CCTGGTTTGTAAAACTCAATTATGG | +120 to +96 | 347 |
| 15S^4^ | GCTTAAACAGCGTGGTCTACA | -57 to -37 | TGCCAATTATATAAACAATTAACCAA | +48 to +23 | 207 |

^1^ The forward M13 sequencing tag (5’-GTAAAACGACGGCCAGT-3’) was added on the 5’ end of all forward

primers.

^2^ Number of nucleotides upstream of the 5’ end of the exon (-) or downstream of the 3’ end of the exon (+).

For exons 1 and 15, the end of the UTR was used rather than the end of the exon.

^3^ The reverse M13 sequencing tag (5’- AACAGCTATGACCATG-3’) was added on the 5’ end of all reverse

primers.

^4^ Primers closer to the intron/exon boundary were used on some formalin-fixed paraffin-embedded tumor samples

for which the amplification of PCR products greater than ~300bp failed, which suggested fragmentation of DNA.
