# Supplementary material for: Loss-of-Function Mutations in PTPN11 Cause Metachondromatosis, but Not Ollier Disease or Maffucci Syndrome
Source: PLoS Genet. 2011 Apr 14;7(4):e1002050. doi: 10.1371/journal.pgen.1002050 (PMC3077396; doi:10.1371/journal.pgen.1002050)
Supplement: Table S7 — Probes used for MLPA. (DOC) [file pgen.1002050.s013.doc]

**Table S7. Probes used for MLPA**

| **Gene** | **Chr** | **Amplicon size (bp)** | **Upstream hybridizing sequence** | **Downstream hybridizing sequence** |
| --- | --- | --- | --- | --- |
| *EXT1*  exon1 middle | 8 | 116 | 5’_GGACACATGCAGGCCAAAAAACGCTA_3’ | 5’_TTTCATCCTGCTCTCAGCTGGCTCTTGTCTCGCCCTTTTGTTTTCATG_3’ |
| *EXT1*  exon1-intron1 | 8 | 112 | 5’_GCATGGCAAAGACTGGCAAAAGCACAAGGAT_3’ | 5’_TCTCGCTGTGACAGAGACAACACCGAGTATGAGAAGTAA_3’ |
| *RGAG1* | X | 102 | 5’_TGACTGTGAATTCTACAACATATGGAGCGC_3’ | 5’_CTACCTTGTGCAAGGCATTGTGGGTAAGTC_3’ |
| *AMMECR1* | X | 106 | 5’_GCTCCCGGAAGATGGTGGTGTCAGCAGAGATG_3’ | 5’_TGCTGCTTTTGCTTCGATGTGCTCTACTGTCA_3’ |
| *SERPINB2* | 18 | 122 | 5’_CATGACTCCAGAGAACTTTACCAGCTGTGGGTTCATGCA_3’ | 5’_GCAGATCCAGAAGGGTAGTTATCCTGATGCGATTTTGCAGG_3’ |
| *CHRDL1* | X | 126 | 5’_CCAATGGAAAGACCTATTCTCATGGCGAGTCCTGGCACCCAA_3’ | 5’_ACCTCCGGGCATTTGGCATTGTGGAGTGTGTGCTATGTACTT_3’ |
