# Supplementary material for: Loss-of-Function Mutations in PTPN11 Cause Metachondromatosis, but Not Ollier Disease or Maffucci Syndrome
Source: PLoS Genet. 2011 Apr 14;7(4):e1002050. doi: 10.1371/journal.pgen.1002050 (PMC3077396; doi:10.1371/journal.pgen.1002050)
Supplement: Text S1 — Supporting Materials and Methods. (DOC) [file pgen.1002050.s014.doc]

**Materials and Methods S1**

**Illumina data analysis.** The amplified targeted array captured libraries were sequenced using two lanes of an Illumina GA II, using single end 80 bp (first array) or 42 bp (second array) sequencing. Novobarcode software (<http://novocraft.com/>) was used to sort the reads according to their 3 bp barcode. For the first capture array, Novoalign was used to align the reads to the reference genome (hg19), using default settings but including adapter trimming. For the second capture array, the BWA aligner (<http://bio-bwa.sourceforge.net/>) was used. PCR duplicates were removed by the MarkDuplicates command in Picard (<http://picard.sourceforge.net/index.shtml>).Variants were called using SAMtools and low confidence variants were removed by the SAMtools varFilter command and by removing all insertions and deletions present in only one read. Annovar (<http://www.openbioinformatics.org/annovar/>) was used to identify coding variants, classify SNPs as synonomous or nonsynonomous, and classify SNPs as either novel or previously described in dbSNP version 132. For each individual, the percentage of reads that overlapped with regions targeted by the capture array was calculated using Galaxy (<http://main.g2.bx.psu.edu/>). The read depth for each base targeted by the array was calculated using the DepthOfCoverage command in the Genome Analysis Toolkit (<http://www.broadinstitute.org/gsa/wiki/index.php/ The_Genome_Analysis_Toolkit>).

**Copy number analysis based on Illumina read depth.** The regions targeted by the capture array were divided into non-overlapping 50 bp windows. For each individual, the read depth for each window was normalized based on the total number of reads obtained for that individual. For each window in an individual, the normalized read depth was divided by the average read depth for that window in all 10 individuals, and the log2 value was calculated. Windows with an average read depth of less than 5X were excluded from the analysis. A perl script was written to find regions of consecutive windows that had log2 ratios below -0.7 or above 0.5, to identify deletions and duplications, respectively.

**Sanger sequencing of *PTPN11****.* All coding *PTPN11* exons were PCR amplified using primers containing an M13 sequencing tag (Table S4). PCR Primers were located between 26 and 160 bp away from the intron/exon boundary. For fragmented DNA extracted from formalin-fixed paraffin-embedded (FFPE) tumor samples, primers closer to the intron/exon boundary were used to allow for smaller PCR products (Table S4). PCR amplification was performed using FastStart Taq DNA Polymerase (Roche) at 95 ºC for 4 min, 35 to 40 cycles of 95 ºC for 30 sec, 60 ºC for 30 sec and 72 ºC at 50 sec, followed by 5 min at 72 ºC. PCR products were sent for purification and sequencing in both forward and reverse directions, using M13 sequencing primers, at the Molecular Genetics Core Facility at Children’s Hospital Boston.

**DNA extraction from exostoses and subcloning of PCR products.** DNA was extracted from several sections of an formalin fixed, paraffin embedded exostosis excised from participant A-IV-5, using a QIAamp DNA FFPE Tissue Kit (Qiagen). To isolate DNA from the cartilage core of this lesion, Laser Capture Microdissection (LCM) was performed. 7-micron sections were placed on PEN membrane glass slides (MDS Analytical Technologies). Sections were stained using Toluidine Blue. The cartilage cores of several sections were isolated using LCM, pooled, and DNA was extracted using a PicoPure DNA Extraction Kit (Applied Biosystems) with an overnight incubation at 65 ºC. A second exostosis, that had been removed from participant A-IV-8, was frozen and embedded in several blocks of OCT. Hematoxylin & Eosin staining of sections of this exostosis was used to identify a block consisting of >90% cartilage cells, and similarly to identify a block consisting of a majority of bone and fibrous tissue. For both blocks, several sections were pooled and DNA was extracted using the PicoPure DNA Extraction Kit (Arcturus) with a 3 hr incubation at 65 ºC. PCR amplification of DNA from both participant’s exostoses was performed using 2 to 6 µl of extracted DNA, in a 20 µl PCR reaction, using PCR conditions described above, and primer pair 4S1 (Table S4), or, to amplify the SNP in intron 11 of *PTPN11,* the primers 5’-GACCCTACAGCACTGCCATT-3’ and 5’-CCCCACCACCTCAATACCTA-3’. For the exostosis from participant A-IV-8, PCR amplimers from the cartilage or the bone and fibrous tissue sections were subcloned into a PCR-4-TOPO Vector using a TOPO TA cloning kit (Invitrogen). Individual colonies were selected and resuspended in a PCR mix, and amplified using primer pair 4S1 (Table S4). To distinguish between mutant and wild-type subclones, the PCR amplimers were restriction digested by combining 0.2 µl of *DdeI*, 1.0 µl of Buffer 3 (New England Biolabs), and 9 µl of each PCR reaction and then incubating at 37 °C for 2 hours. Digested products were separated by electrophoresis on a 4% agarose gel. The 5 bp deletion creates a novel DdeI restriction site.

**Multipex Ligation-Dependent Probe Amplification.** MLPA was performed using the SALSA MLPA EK1 Kit (MRC-Holland). A custom probemix was used, including two probes targeting exon 1 of *EXT1* [1,2] and four control probes (Table S7). The resulting labeled PCR products were separated according to size (Applied Biosystems 3730 DNA Analyzer) and the height of each peak was calculated. For each *EXT1* peak, a ratio was calculated by dividing the height of the *EXT1* peak by the average height of the non-*EXT1* peaks. For each participant, this ratio was divided by the average ratio obtained in the control samples, to obtain a corrected ratio, such that a value of 1.0 represents a copy number of 2, and a value below the threshold of 0.80 represents a deletion.

**Microarray-based comparative genomic hybridization analysis.** To determine if some patients with MC had deletions or duplications within the candidate interval on chromosome 12, we performed microarray-based Comparative Genomic Hybridization (aCGH) on 8 affected individuals from 7 families (Table S1). We designed an 8x60K Agilent SurePrint G3 array using eArray to include probes spanning the linkage interval, with an average probe spacing of 160 bp. We found 7 potential small duplications or deletions, all of which were less than < 1.5 kb in size. One of the duplications spanned a coding region in *IFT81*, but we could not verify this duplication using a PCR-based assay.

**Immunodetection of SHP2.** An excised exostotic lesion from participant B-IV-7 and rib cartilage from an unaffected individual were homogenized in RIPA buffer (25 mM Tris pH7.4, 150 mM NaCl, 5 mM EDTA, 1% NP-40, 0.1% SDS, 0.5% sodium deoxycholic acid). HELA cells were lysed directly in NuPAGE LDS sample buffer (Invitrogen). Protein extracts were separated on a NuPAGE 4-12% Bis-Tris Gel (Invitrogen) and transferred to an Invitrolon PVDF membrane (Invitrogen). Immunodetection of SHP2 was performed using a WesternBreeze Chemiluminescent Immunodetection Kit (Invitrogen) and a 1:1000 dilution of mouse-anti-SHP2 (610621, BD Biosciences). As a loading control, immunodetection using mouse-anti-actin antibody (ab3280, Abcam, 1:600 dilution), was performed on the same blot.

**Supplemental References**

1. White SJ, Vink GR, Kriek M, Wuyts W, Schouten J, et al. (2004) Two-color multiplex ligation-dependent probe amplification: detecting genomic rearrangements in hereditary multiple exostoses. Hum Mutat 24: 86-92.

2. Jennes I, Entius MM, Van Hul E, Parra A, Sangiorgi L, et al. (2008) Mutation screening of EXT1 and EXT2 by denaturing high-performance liquid chromatography, direct sequencing analysis, fluorescence in situ hybridization, and a new multiplex ligation-dependent probe amplification probe set in patients with multiple osteochondromas. J Mol Diagn 10: 85-92.
